# Tauopathy in the young autistic brain: novel biomarker and therapeutic target

**DOI:** 10.1038/s41398-020-00904-4

**Published:** 2020-07-13

**Authors:** Iris Grigg, Yanina Ivashko-Pachima, Tom Aharon Hait, Vlasta Korenková, Olga Touloumi, Roza Lagoudaki, Anke Van Dijck, Zlatko Marusic, Mirna Anicic, Jurica Vukovic, R. Frank Kooy, Nikolaos Grigoriadis, Illana Gozes

**Affiliations:** 1 Elton Laboratory for Neuroendocrinology, Department of Human Molecular Genetics and Biochemistry, Sackler Faculty of Medicine, Sagol School of Neuroscience and Adams Super Center for Brain Studies, Tel Aviv University, Tel Aviv, Israel; 2 The Blavatnik School of Computer Science, Tel Aviv University, Tel Aviv, Israel; 3 BIOCEV, Institute of Biotechnology CAS, Průmyslová 595, 252 50 Vestec, Czech Republic; 4 Department of Neurology, Laboratory of Experimental Neurology, AHEPA University Hospital, Aristotle University of Thessaloniki, Thessaloniki, Greece; 5 Department of Medical Genetics, University of Antwerp, Antwerp, Belgium; 6 Clinical Department of Pathology and Cytology, University Hospital Centre Zagreb, Zagreb, Croatia; 7 Department of Pediatrics, Division of Pediatric Gastroenterology, Hepatology and Nutrition, University Hospital Centre Zagreb, School of Medicine, Zagreb, Croatia

**Keywords:** Diagnostic markers, Diagnostic markers

## Abstract

Given our recent discovery of somatic mutations in autism spectrum disorder (ASD)/intellectual disability (ID) genes in postmortem aged Alzheimer’s disease brains correlating with increasing tauopathy, it is important to decipher if tauopathy is underlying brain imaging results of atrophy in ASD/ID children. We concentrated on activity-dependent neuroprotective protein (ADNP), a prevalent autism gene. The unique availability of multiple postmortem brain sections of a 7-year-old male, heterozygous for ADNP de novo mutation c.2244Adup/p.His559Glnfs*3 allowed exploration of tauopathy, reflecting on a general unexplored mechanism. The tested subject exhibited autism, fine motor delays, severe intellectual disability and seizures. The patient died after multiple organ failure following liver transplantation. To compare to other ADNP syndrome mutations, immortalized lymphoblastoid cell lines from three different patients (including ADNP p.Arg216*, p.Lys408Valfs*31, and p.Tyr719* heterozygous dominant mutations) and a control were subjected to RNA-seq. Immunohistochemistry, high-throughput gene expression profiles in numerous postmortem tissues followed. Comparisons to a control brain and to extensive datasets were used. Live cell imaging investigated Tau-microtubule interaction, protecting against tauopathy. Extensive child brain tauopathy paralleled by multiple gene expression changes was discovered. Tauopathy was explained by direct mutation effects on Tau-microtubule interaction and correction by the ADNP active snippet NAP. Significant pathway changes (empirical *P* value < 0.05) included over 100 genes encompassing neuroactive ligand–receptor and cytokine–cytokine receptor interaction, MAPK and calcium signaling, axon guidance and Wnt signaling pathways. Changes were also seen in steroid biosynthesis genes, suggesting sex differences. Selecting the most affected genes by the ADNP mutations for gene expression analysis, in multiple postmortem tissues, identified Tau (MAPT)-gene-related expression changes compared with extensive normal gene expression (RNA-seq) databases. *ADNP* showed relatively reduced expression in the ADNP syndrome cerebellum, which was also observed for 25 additional genes (representing >50% of the tested genes), including *NLGN1, NLGN2, PAX6, SMARCA4*, and *SNAP25*, converging on nervous system development and tauopathy. NAP provided protection against mutated ADNP disrupted Tau-microtubule association. In conclusion, tauopathy may explain brain-imaging findings in ADNP syndrome children and may provide a new direction for the development of tauopathy protecting drug candidates like NAP in ASD/ID.

## Introduction

Recent whole-exome sequencing studies in autism spectrum disorder (ASD)/intellectual disability (ID) cohorts, found ADNP^1,2^ as one of the most frequently de novo mutated genes, responsible for about 0.2% of ASD cases^3–5^. ADNP syndrome, also known as Helsmoortel–Van der Aa syndrome (OMIM identification 615873, Orphanet—https://www.orpha.net/consor/cgi-bin/OC_Exp.php?lng=EN&Expert=404448), is a complex developmental disorder with robust neurological consequences and effects on multiple organ functions. ADNP syndrome patients share multiple clinical features^3,6^.

ADNP is imperative for brain development and normal function by regulating multiple key genes associated with synaptic transmission including tubulin/microtubules, ion channel, and autophagy-controlling genes^7,8^. In addition to its suggested role as a transcription factor and chromatin remodeling protein, in the neuronal cytoplasm^9^, ADNP interacts directly with cytoplasmic proteins regulating basic cellular processes. Thus, it was identified as a vital gene for the microtubule network essential for synapse formation^10^ and maintaining normal axonal transport rates^7^.

Importantly and highly pertinent to the current investigation, ADNP contains a neuroprotective motif, a drug candidate NAP^1^ (davunetide, CP201). This neuroprotective motif contains eight amino acids, NAPVSIPQ, with SIP (SxIP) being the signature structure for interaction with microtubule end binding proteins, enhancing dendritic spine formation^11^, enlisting Tau to the microtubule shaft^12,13^, thus protecting microtubule-dependent axonal transport^14^ and inhibiting Tau pathology^15–17^. As such, *Adnp* haploinsufficiency in mice results in tauopathy, which is rescued by NAP treatment and is exacerbated with aging^15^.

Here, having postmortem tissues from 7-year-old male, heterozygous for an ADNP de novo mutation c.2244Adup/p.His559Glnfs*3, we concentrated on a potential tauopathy outcome in this young ADNP subject. Furthermore, using RNA-seq on EBV-transformed human lymphoblastoid cell lines from a healthy control and from three ADNP syndrome patients, carrying three different mutations, we discovered ADNP-dependent altered gene regulation that was also mimicked by our *Adnp*-deficient mouse model^10,18^ and was correlated with the finding of this very early onset tauopathy. Our previous data, in cell cultures showed that ADNP mutations (p.Tyr719*, p.Arg730*) reduced microtubule–Tau interaction^19^ (leading to tauopathy)^13,16,19^, which was rescued by NAP treatment^19^. We now extended this finding to shorter ADNP mutated proteins (p.Ser404*) discovering a significant reduction in microtubule–Tau interaction (reflected in increases in immobile Tau) and complete reversal by NAP treatment.

## Methods

### ADNP postmortem case

Clinical information of a deceased 7-year-old ADNP syndrome boy was received from the mother (caregiver) and tending clinicians, under informed consent. Under informed consent from the mother (caregiver of the deceased child), postmortem tissues were collected during autopsy and documented with half undergoing immediate freezing (−180 °C) and half fixation for further histochemical analyses^7^. Specifically, for RNA extractions, tissue samples were surgically removed and frozen in vials (brain stem, cerebellum, hippocampus, pituitary (hypophysis), striatum, visual cortex, blood, colon, muscle (gastrocnemius, tibialis, and tongue), heart, kidney, pancreas, skin, spinal cord, stomach, testis, and thyroid. Each tissue was homogenized using Bullet Blender^®^ and the appropriate beads.

### Control brain

Control hippocampus tissue was obtained by a surgical removal from a 31-year-old subject, fixed and submitted to further histological procedures.

### Histology

Human paraffin sections (6 μm) were deparaffinized and hydrated in xylene and alcohol solutions, rinsed with distilled water. The sections were then stained with Gallyas technique in order to identify Tau pathology.

### Immunohistochemistry

Standard methods and antibodies are described in the Supplementary methods.

### Neuronal-like cell models, differentiation, co-transfection of overexpressing plasmid, and fluorescence recovery after photobleaching (FRAP)^19^

All methodology was published before^19^ and is further detailed in the Supplementary methods.

Protein expressing plasmids were prepared based on a pEGFP-C1 backbone and inclusion of the full-length *ADNP* or of the truncated form expressiong the mutated p.Ser404* ADNP (Supplementary materials).

### Cell RNA extraction and RNA sequencing

RNA was extracted from EBV-transformed human lymphoblastoid cell lines using TRI Reagent^®^, as instructed (Sigma-Aldrich, MO, USA). At the time of extraction, cell confluence was about 70–80%. Cell lines included one healthy control and three ADNP syndrome patients, carrying three different mutations (Table S1). Technical details of cell line library preparation, sequencing, and data analysis are described in the Supplementary methods and published articles^10,18^.

### Organ DNA/RNA extraction

After homogenization, DNA and RNA were extracted using ZR-Duet^TM^ DNA/RNA MiniPrep Plus (Zymo Research. CA, USA). A DNA sample from the kidney was Sanger sequenced to validate the mutation (Supplementary Results, Fig. S1. Note, all supplementary figures are labeled S and the respective Fig. number).

### qPCR gene expression analysis

Analysis was performed in BIOCEV institute (Prague, Czech Republic) with BioMark HD system (Fluidigm, San Francisco, CA) (Supplementary methods)^10^.

### RNA expression data

Normal gene expression data from two extensive databases were used: HPA RNA-seq normal tissues (NCBI) and GTEx portal (accession number phs000424.vN.pN)^20^.

### Bioinformatics

#### Analysis of lymphoblastoid RNA-seq data

We downloaded our preprocessed lymphoblastoid (Table S1) RNA-seq data from gene expression omnibus (GEO) (GSE81268)^10,18^ and computed the log fold change (LFC) as follows: First, for each gene, we computed the median expression value of the three mutated samples (expression-Ig1, expression-SSC4121, and expression-SSC8311). Second, we divided the median expression value with expression-Lympho_cont column and applied log2 (we added a prior value of 1 to the nominator and denominator to prevent dividing by zero or logarithm of zero). Results are available at Supplementary Table S1.

We ranked the LFCs from highest to lowest, and applied the gene set enrichment analysis (GSEA)^21^ implemented in Expander^22^. We chose to apply 1000 permutation tests. Gene sets selected for the analysis are: Kyoto Encyclopedia of Genes (KEGG) pathways gene-sets from EXPANDER and MSigDB (C2 collection) databases^23,24^, Reactome pathways and gene ontology gene-sets from MSigDB (C2 and C5 collections)^24^. Results are available in Supplementary Table S2.

#### Comparison between healthy controls and ADNP syndrome

We applied log_2_(*x* + 1) transformation on the HPA, GTEx, and ADNP syndrome expression values (Table S3) in order to reduce the effect of outliers in the data. Next, for each gene, we created a heatmap using pheatmap R package with standardization (scale = “row” in the “pheatmap” function), per dataset (HPA, GTEx, and ADNP syndrome), of each gene across the tissues (Kolde R. pheatmap: Pretty Heatmaps [Internet]. 2019. Available from: https://cran.r-project.org/package=pheatmap) (Fig. S2). The standardization helped us to compare the expression values between the datasets, per tissue and per gene, since the distributions between the datasets were not the same (Fig. S3).

## Results

### Clinical disease manifestation

Seven-year-old male was diagnosed as a toddler with ASD, confirmed by cDNA sequencing as ADNP syndrome (heterozygous for a de novo mutation c.2244Adup/p.His559Glnfs*3, RefSeq isoform NM_015339.4) (Fig. S1). Medical records showed that besides autistic traits, the patient presented gross and fine motor delays, severe ID and seizures^6^. Specifically, after a first liver transplantation at the age of about 3.5 years, the patient developed generalized symptomatic epilepsy. As epilepsy was refractory, several antiepileptic drugs were used to control it. He was first on carbamazepine, then oxcarbazepine was added. Later carbamazepine was stopped, and he continued on oxcarbazepine and levetiracetam. Following that, he was switched to clobazam and topiramate, and for the last 18 months before death, he had been prescribed a combination of three antiepileptic drugs including: levetiracetram + topiramate + clonazepam. The patient also suffered from low muscle tone, convergence insufficiency (impaired 3D vision), as well as parent-reported feeding/eating and sleep problems. He also manifested a potential ADNP marker, early eruption and loss of deciduous teeth (full set of teeth by the age of one)^18^. The patient died after multiple organ failures following a second liver transplantation. An autopsy was performed and different tissue samples were taken for analysis.

### ADNP syndrome postmortem tauopathy is potentially rescued by NAP treatment

**Fig. 1:**
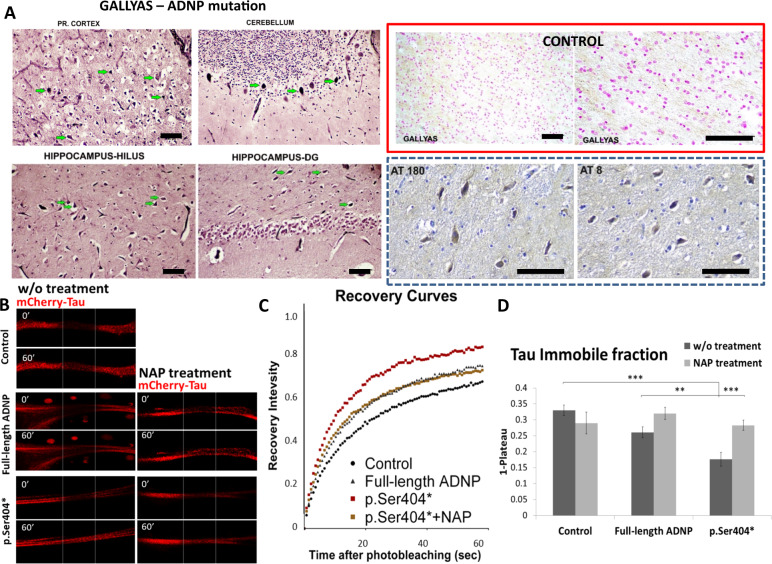
Discovery of ADNP syndrome juvenitle postmortem tauopathy and potential rescue by NAP treatment. **a** The ADNP syndrome juvenile brain exhibits intensive tauopathy. Gallyas staining (Tau pathology) is indicated by green arrows. Frontal cortex (Pr cortex), cerebellum, hippocampal hilus, and dentate gyrus showed positive staining. In the hippocampus the positive cells were in the hillus and in the molecular area, in the granular area of dentate gyrus, CA1, CA2, CA3, there were no positive cells. While no apparent Gallyas staining was observed in the control hippocampus (red box), the ADNP case study tauopathy was verified by hippocampal hyperphosphorylated Tau immunohistochemistry (AT180 and AT8 antibodies). Bars represent 100 μm. The ADNP snippet, drug candidate. **b** NAP enlists Tau to microtubules in the face of ADNP mutations: protection against Tauopathy. Representative images of photo-bleaching (0′) and fluorescence recovery (60′) of mCherry-tagged Tau in differentiated N1E-115 cells co-transfected with GFP-tagged full-length ADNP or truncated p.Ser404* ADNP form with or without NAP treatment (10^−12^M, 4h). Transfection with backbone plasmid (pEGFP-C1) expressing non-conjugated GFP, was performed as a control. **c** Fluorescence recovery after photobleaching (FRAP) recovery curves of normalized data (see “Online Resources 1”). **d** The graph represents averages (±SEM) of the fitted data (from three independent experiments) of immobile fractions. Normalized FRAP data were fitted with one-exponential functions (GraphPad Prism 6) and statistical analyses was done by Two Way ANOVA (SigmaPlot 11, with all pairwise multiple comparison procedures—Tukey Test). Statistical significance is presented by **P < 0.01, ***P < 0.001. Control *n* = 64; control+NAP *n* = 14; full-length ADNP *n* = 55; full-length ADNP+NAP *n* = 46; p.Ser404* *n* = 33; p.Ser404* + NAP *n* = 62.

The combination of histochemical methods (Fig. 1a, Gallyas staining, frontal cortex, cerebellum hippocampal hilus, and dentate gyrus, and Fig. S4, hypothalamus and olfactory bulb) and immunohistochemistry (hippocampal hilus and dentate gyrus, with antibodies recognizing Alzheimer’s disease-related hyperphosphorylated Tau AT8 and AT180, Fig. 1a, dashed blue box)^15^ identified extensive tauopathy. However, the corpus callosum and the trigeminal nerve were negative (Fig. S4). No apparent staining was observed in the control brain hippocampus (Fig. 1a red box, “GALLYAS” staining).

Our previous data, in cell cultures showed that ADNP mutations (p.Tyr719*, p.Arg730*) reduced microtubule–Tau interaction^19^ (leading to tauopathy)^13,16,19^, which was rescued by NAP treatment^19^. Here, we extended this finding to shorter ADNP mutated proteins (p.Ser404*, Fig. S5A,B) discovering significant reduction in microtubule–Tau interaction (reflected in increases in immobile Tau content) and complete reversal by NAP treatment (Fig. 1b, c).

At the immunohistochemical level, two additional findings were observed. (1) A dramatic hippocampal staining of postsynaptic density protein 95 (PSD95). (2) A similar increased stain of the N-methyl-D-aspartate (NMDA) receptor 1 (NMDAR1) in the hippocampus (Fig. S6A), which was much lower in the control hippocampal stain (albeit in a different hippocampal region—a control seizure-free region, Fig. S6B).

### ADNP mutant lymphoblasotoid RNA-Seq supporting Tau pathology

To evaluate human mutated ADNP (different mutations including p.Arg216*, p.Lys408Valfs*31, and p.Tyr719*) signature RNA expression patterns, we first looked at our lymphoblatoids RNA-seq datasets (“Methods” and Table S1). Three ADNP mutated samples were sufficient for our analysis as most of the genes had similar expression values in two out of the three samples. Therefore, we chose for each gene, the median expression value in the three samples as the representative gene expression for the mutated ADNP samples (“Methods”). We applied a GSEA on the LFCs between the ADNP mutated and control gene expressions (GSEA results are available in Table S2). We discovered effects on multiple pathways. Significant KEGG pathway changes (empirical *P* value < 0.05) encompassing over hundred genes included the following, neuroactive ligand–receptor and cytokine–cytokine receptor interaction (implicated for ADNP signaling)^25,26^, MAPK signaling, calcium signaling (implicated in ADNP brain activity)^7^, axon guidance and Wnt signaling pathway (previously associated with ADNP regulation of teeth/bone and brain)^18^. Highly significant changes were also seen in steroid biosynthesis genes, suggesting sex differences, as noted in mice and linked with microtubule regulation^7^. Interestingly, Alzheimer’s disease and Parkinson’s disease were among the discovered KEGG pathways (with over a 100 genes of interest, however with empirical *P* value 0.093/0.12, respectively). We also observed neuron-related GO biological processes enrichments such as negative regulation of neurological system process, forebrain generation of neurons, and neuroepithelial cell differentiation (empirical *P* value < 0.001).

**Fig. 2:**
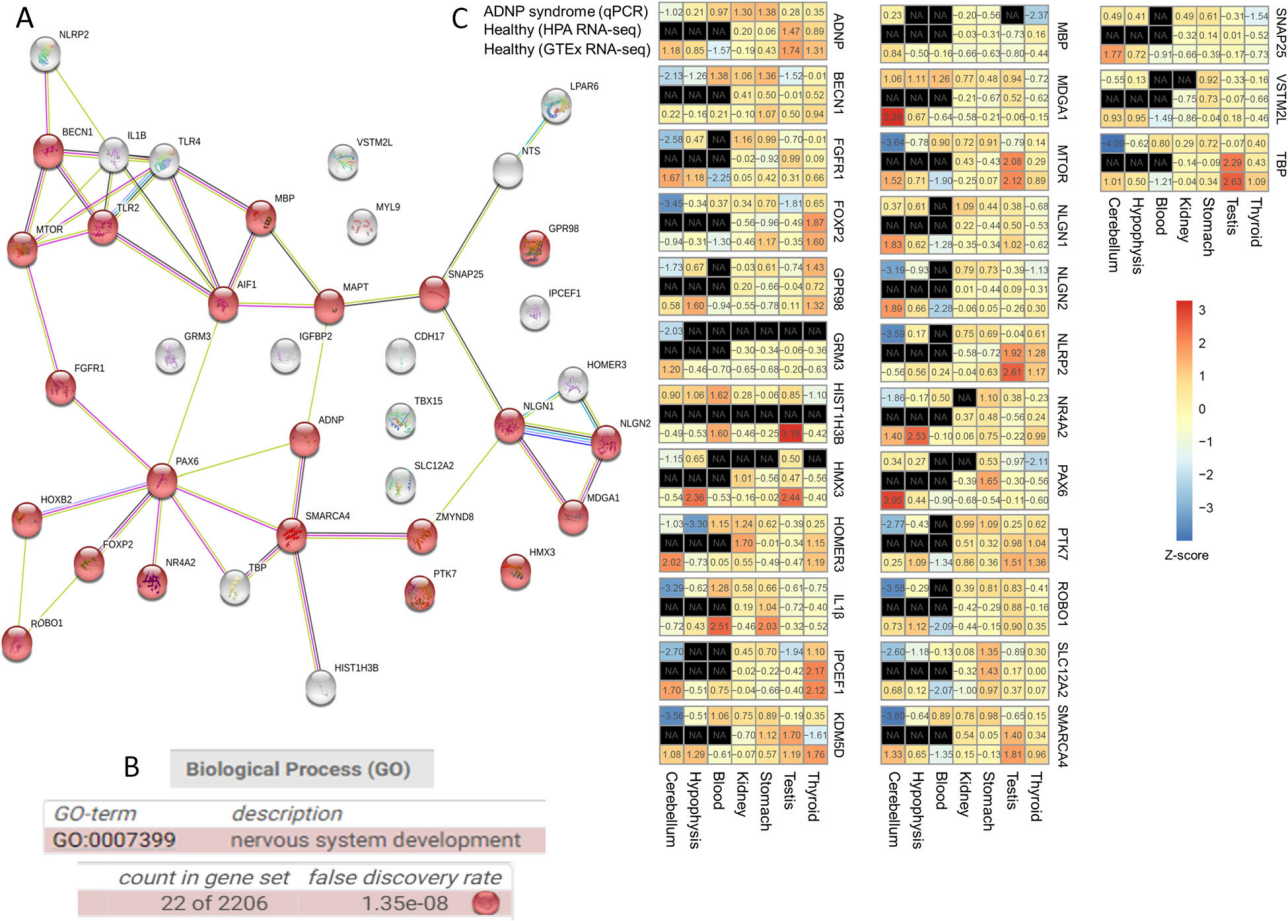
Significant ADNP influence on protein expression associated with signature transcript changes because of ADNP mutation. Gene transcripts (Tables 1 and 2) were subjected to String protein interaction analysis (https://string-db.org/cgi/network.pl?taskId=J365DTd906k7) including MAPT – Tau, which was found to be hyperphosphorylated, forming neurofibrillary tangles in Adnp-haploinsufficient mice^15^ and above (**a**). Expected enrichment/involvement in the biological process of nervous system development (**b**). **c** Heatmaps of selected genes in a subset of tissues for examination. We examined 27 genes in cerebellum, hypophysis, blood, kidney, stomach, testis, and thyroid. The heatmaps were extracted from Online Resource 5. Each triplet of rows represents a single gene in three datasets (see the ADNP gene example on the top-left plot): ADNP syndrome (qPCR), Healthy (HPA RNA-seq), and Healthy (GTEx RNA-seq). Expression values (z-scores) are indicated within the cells. Gene transcripts showing significant reduction in the cerebellum include: Beclin1 (*BECN1*), fibroblast growth factor receptor 1 (*FGFR1*), forkhead box protein P2 (*FOXP2*), G-protein coupled receptor 98 (*GPR98*), glutamate metabotropic receptor 3 (*GRM3*), homeobox 3 (*HMX3*), homer scaffold protein 3 (*HOMER3*), interleukin 1 beta (*IL1beta*), interactor protein for cytohesin exchange factors 1 (*IPCEF1*), Lysine-specific demethylase 5A (*KDM5D*), myelin basic protein (*MBP*), MAM domain-containing glycosylphosphatidylinositol anchor protein 1 (*MDGA1*), mammalian target of rapamycin (*MTOR*), neuroligin1/2 (*NLGN1, NLGN2*), nuclear receptor family pyrin domain containing (*NLRP2*), nuclear receptor subfamily 4 group A member 2 (*NR4A2*), paired box 6 (*PAX6*), inactive tyrosine-protein kinase 7 (*PTK7*), roundboat guidance receptor (*ROBO1*), solute carrier family 12 member 2 (*SLC12A2*, electrically silent transporter system, mediates sodium and chloride reabsorption), SWI/SNF related, matrix associated, actin dependent regulator of chromatin, subfamily A, member 4 (*SMARCA4*) also known as Brahma protein-like 1 (*BRG1*), synaptosomal nerve-associated protein 25 (*SNAP25*), TATA-box-binding protein (*TBP*) and V-set and transmembrane domain-containing protein 2-like protein (*VSTM2L*).

Table 1, prepared based on our RNA-seq data (GEO, GSE81268)^10,18^ lists the genes that showed the highest change when comparing ADNP mutated lymphoblastoid cells to a control lymphoblastoid cell line. Table 2 lists additional genes of interest that changed either as consequence of *Adnp* deficiency or as a results of NAP treatment or of both. These genes/proteins differentiate ADNP syndrome and healthy control lymphoblastoids^10,18^. As expected, String analysis for protein–protein interactions showed that most of the genes regulated by ADNP (i.e., genes changing in expression presumably because of ADNP mutation as explained in Table 2) exhibited a connection to Tau (MAPT) and the expected nervous system development pathway enrichment (Fig. 2).

**Table 1:** RNA SEQ data (GEO, GSE81268)^10,18^.

Gene*	Fold change (Ig1 vs. Lympho)	Fold change (SSC4121 vs. Lympho)	Fold change (SSC8311 vs. Lympho)	Expression-Lympho_cont	Expression-Ig1	Expression-SSC4121	Expression-SSC8311
ROBO1	745.647	91.2253	812.44	0.0128824	9.60575	1.1752	10.4662
NLRP2	112.939	124.576	905.512	0.0184944	2.08874	2.30397	16.7469
HOXB2	107.756	94.6583	91.1358	0.0847141	9.12846	8.0189	7.72049
IPCEF1	73.9769	49.2174	89.7259	0.219583	16.2441	10.8073	19.7023
PTK7	72.7466	22.561	122.293	0.0228934	1.66542	0.516499	2.79971
TLR4	72.0192	91.7155	63.094	0.0117378	0.845345	1.07654	0.740583
TBX15	63.2896	212.859	167.082	0.0388303	2.45756	8.26537	6.48786
ALOX12P2	52.9232	58.8549	52.5783	0.0490289	2.59477	2.88559	2.57786
TLR2	49.104	83.3778	275.744	0.00997296	0.489712	0.831523	2.74999
IGFBP2	44.1936	12.5067	5.84204	0.0949699	4.19706	1.18776	0.554818
LPAR6	34.7975	42.8991	35.26	0.441516	15.3636	18.9406	15.5678
AIF1	12.5361	8.50887	16.9555	0.156669	1.96403	1.33308	2.65641
FGFR1	6.50824	6.67026	6.91029	0.753597	4.90459	5.02669	5.20757
VSTM2L	−6.46587	−7.61594	−27.1737	2.19728	0.339827	0.28851	0.0808603
GRM3	−17.1065	−5.70896	−11.9821	0.446002	0.026072	0.0781232	0.0372223
LOC151174	−29.3255	2.94275	−4.56462	0.554209	0.0188985	1.6309	0.121414
GPR98	−31.1584	−4.95165	−21.8246	0.179003	0.00574493	0.0361501	0.00820188
HOMER3	−36.3133	−19.0099	−135.655	4.0601	0.111807	0.213578	0.0299296
MYL9	−64.4865	−42.5937	−19.0713	37.8958	0.587655	0.889704	1.98705
HMX3	−75.6048	−7.00876	−2.882	10.4248	0.137885	1.4874	3.61722
CDH17	No change	−95.1494	No change	2.49733	0	0.0262464	0

(*) Here, unlike the fold changes computed for GSEA analysis (“Methods”), we ranked genes by the highest fold change among the three mutated samples versus the control “Lympho” sample.

**Table 2:** Gene selection rationale.

Gene and reason for choice	Fold change (Ig1 vs. Lympho)	Fold change (SSC4121 vs. Lympho)	Fold change (SSC8311 vs. Lympho)	Expression-Lympho_cont	Expression-Ig1	Expression-SSC4121	Expression-SSC8311
HIST1H3B	2.728	4.90457	4.67363	0.143763	0.392185	0.705095	0.671894
Reason	Decreased in the cortex of Adnp^+/−^ female mice and increased (normalized) with NAP CP201 treatment^10^						
SLC12A2	1.48183	1.64404	1.67656	3.31149	4.90706	5.44423	5.55191
Reason	(Slc12a2, Slc9a3) showed ADNP-age-dependent regulation in the hippocampus, cortex and spleen in mice^10^						
ADNP	1.34291	1.29374	1.31103	28.3059	38.0124	36.6205	37.1097
Reason	Studied gene						
NR4A2	1.27615	1.1638	1.109	0.566908	0.723459	0.659766	0.628699
Reason	Nuclear receptor subfamily 4 group A member 2—pathogenic NR4A2 variants cause developmental delays/intellectual disabilities DD/ID and/or autism spectrum disorder (ASD) (https://panelapp.genomicsengland.co.uk/panels/285/gene/NR4A2/), regulated by ADNP^27^						
SNAP25	1.2276	1.10353	−3.42346	0.124965	0.153407	0.137903	0.0365025
Reason	SNAP-25 regulates dendritic spine formation through postsynaptic binding to p140Cap^39^ that in turn interacts with ADNP/NAP (CP201)^11^						
MBP	1.08443	−1.10046	1.3582	17.4361	18.9082	15.8444	23.6817
Reason	Myelin basic protein – ADNP is associated with myelin – white matter – health^40^						
SMARCA4	1.04815	−1.15125	−1.0588	26.9341	28.2311	23.3955	25.4384
Reason	SMARC4, also known as BRG1 is an ADNP, SWI/SNF-interacting protein^32,41^						
TBP	1.02512	1.01825	1.13606	10.2996	10.5583	10.4876	11.7009
Reason	Used sometimes for calibration as house-keeping gene						
NLGN2	−1.11457	−1.43361	1.35016	0.328901	0.295093	0.229422	0.44407
Reason	Linked with synaptic regulation, shows age-dependent hippocampal reduction in Adnp^+/−^ mice^41^						
NLGN1	not found	not found	not found	not found	not found	not found	not found
Reason	Linked with synaptic regulation, shows hippocampal sex-dependent regulation in Adnp^+/+^ as well as Adnp^+/−^ mice^41^						
MTOR	−1.18542	−1.03791	−1.05758	10.8397	9.14418	10.4438	10.2495
Reason	Autophagy-related, regulated by Adnp^+/−^ and corrected by NAP (CP201) in the mouse spleen^10^						
BECN1	−1.28996	−1.1749	−1.14578	32.6192	25.287	27.7633	28.4691
Reason	Regulated by Adnp/NAP (CP201)^7,8,10^						
IL1B	−1.62136	−3.90792	1.32081	0.520924	0.321288	0.1333	0.688041
Reason	Regulated by the Adnp^+/−^ genotype^7,10^						
MDGA1	−1.62457	−238.553	−1.43052	1.78268	1.09732	0.00747288	1.24618
Reason	MAM domain containing glycosylphosphatidylinositol anchor 1, associated with nervous system development and psychiatric disorders https://www.ncbi.nlm.nih.gov/gene/266727						
PAX6	−2.11482	−1.09227	1.2463	0.138463	0.0654726	0.126766	0.172566
Reason	Important for nervous system development, regulated by ADNP^42^						
KDM5D	Not determined change	Not determined change	Not determined change	0	15.1514	0	13.5382
Reason	Increased in the Adnp^+/−^ mouse spleen, decreased (normalized) by NAP (CP201) treatment^10^						
NTS	Not determined change	Not determined change	Not determined change	0	0.0298807	0	2.43162
Reason	This gene encodes a common precursor for two peptides, neuromedin N and neurotensin and may function as a neurotransmitter or a neuromodulator—was discovered as regulated by ADNP^27^						
FOXP2	No change	−4.58755	−3.8514	0.0431349	0	0.0094026	0.0111998
Reason	Important for language regulated by ADNP and NAP (CP201)^33,43^

In search for signature transcriptome associated with the ADNP syndrome in the postmortem samples, RNA extracted from multiple tissues was submitted to high throughput qPCR^10^ focusing on the chosen transcripts (Tables 1 and 2, primer sets, Table S4) and compared with normal expression (obtained from two databases, “Methods”). Since each of the public databases and our qPCR results were obtained and calculated with different methods, we applied a log_2_ transformation and a standardization scheme, per gene and per dataset (Methods). Below, we examined genes mostly showing high and significant Spearman correlation between the GTEx and HPA datasets (Fig. S7; Spearman *P* value < 0.1). We also considered genes with limited expression values in one of the control datasets (HPA or GTEx) and expressed in at least 15 out of 24 examined tissues in the other control and ADNP syndrome datasets. This way, we were confident that the standardization of the gene expression across tissues per dataset represents the relative gene expression between the tissues per dataset.

ADNP showed relatively reduced expression in the child’s postmortem cerebellum (Fig. S2). This relatively reduced expression was also observed for *BECN1, FGFR1, FOXP2, GPR98, GRM3, HMX3, HOMER3, IL1beta, IPCEF1, KDM5D, MBP, MDGA1, MTOR, NLGN1, NLGN2, NLRP2, NR4A2, PAX6, PTK7, ROBO1, SLC12A2, SMARCA4, SNAP25, TBP*, and *VSTM2L* (see Fig. 2 legend for complete gene names). All these genes converge on nervous system development and represent a majority of the tested genes. Interestingly, while expression in testis, pituitary (hypophysis) and thyroid was occasionally similar in trend with the cerebellum, the stomach, kidney and blood exhibited occasionally opposite (increased) trends in gene expression associated with the ADNP mutation (Fig. 2, Fig. S2).

In contrast to the listed gene transcripts above, histone cluster 1 H3 family member B (*HIST1H3B*) that encodes a nucleosome core protein, showed a relatively high expression in the ADNP syndrome cerebellum and pituitary (hypophysis), and a decreased expression in the hippocampus, indicating gene specificity.

## Discussion

The novel tauopathy findings in ASD/ID are directly related to ADNP function that by binding to microtubule end binding proteins, which in turn bind to Tau, enhances Tau association with microtubules^12^ and protects against tauopathy^15^. However, in the *Adnp*^*+/−*^ mice tauopathy appeared with aging^15^, similar to Alzheimer’s disease^19^ and unlike the childhood discovery here. Interestingly, ADNP/NAP preferentially interact with the alternatively spliced three microtubule binding repeat dynamic Tau (associated with development) compared with four microtubule binding repeat Tau^17^ implicating potential early onset tauopathy in association with the ADNP syndrome. In this respect, early Tau hyperphosphorylation was observed in the *Adnp*^*+/−*^ mice, which was reversed by NAP treatment^15^.

The ADNP-mutation associated PSD95 and NMDAR1 increased expression might be related to aberrant synaptic plasticity as observed in the *Adnp*^+/−^ mice also at the dendritic spine level^10^ and to increased excitotoxicity linked with seizures, which have been observed in the case study, with intact ADNP/NAP, protecting against excitotoxicity^1^.

*ADNP* expression was reduced in the case study cerebellum, hypophysis, testis, thyroid, and skin because of ADNP mutation (Fig. S2), with some other tissues such as stomach, kidney and blood showing an apparent increase (as partly noted above; Fig. 2). In this respect, ADNP, regulating its own expression^27^, has been implicated in brain function (ID) affecting all known ADNP syndrome cases^6^ with motor impediments correlated in severity to ID, endocrine problems (24.5% of the children/thyroid hormone problem, 15%), early puberty (30%), skin problems, gastrointestinal problems (83% of the children), and urogenital problems (28%)^6^.

*PAX6*, a major regulatory gene for brain development, originally discovered by us as a major ADNP-regulated gene during embryonic and postnatal development^15,27^ showed here a dramatically decreased expression in the cerebellum in the ADNP case compared with controls (as noted above; Fig. S2). This finding is in-line with known functions of PAX6 and the ADNP phenotype, with PAX6 playing a seminal role in the development of glutamatergic neurons^28^ and ADNP regulating the glutamatergic synapse^29^. The seemingly robust control of brain-development associated gene expression (Fig. 2) is now further exemplified in the human brain PAX6 regulation. PAX6 has a key role in neural tissue development, particularly the eye. Mutations in this gene are associated with ocular disorders, cerebral malformations auditory deficiency, pancreatic and pituitary development and function, development of the CNS, and beyond^30,31^.

SMARCA4 (BRG1), an ADNP-interacting protein^32^ showed here to be regulated by the ADNP mutation (transcript down regulated also in the hypophysis and thyroid as in the ADNP reduced expression) (Fig. S2). SMARCA4 is also implicated in autism^8^. Similarly, BECN1, a major regulator of autophagy, which has been previously shown to be downregulated by *Adnp* deficiency, and corrected by the ADNP snippet drug candidate NAP, microtubule end protein binding motif (SxIP) SKIP^7^, was downregulated here as well.

A closer inspection of brain gene expression showed an overall downregulation of *ADNP* (mimicking *Adnp* haploinsufficiency, except in the hippocampus) and a dramatic downregulation *FOXP2* in the cerebellum. FOXP2, a major transcription factor involved in language acquisition was increased in the hippocampus (Fig. S2), as was seen before also in the haploinsufficient Adnp male mouse hippocampus^33^, with direct association to language acquisition impediments in the ADNP children^6^.

Looking at genes that might be associated with peripheral phenotypes of the ADNP syndrome, *FGFR1* showed reduced expression in the cerebellum and hypophysis. This gene influences mitogenesis as well as differentiation and is important for normal bone development. This particular family member is involved in limb induction and is related to several syndromes in which symptoms include: high forehead, wide-set eyes, wide feet and dental aberrations^34,35^, well matched with the ADNP syndrome phenotype^18^. FGFR1 has a role in neural crest formation as well^36^. *HIST1H3B* that encodes a nucleosome core protein, showed a relatively high expression in the cerebellum and hypophysis, and a decreased expression in the hippocampus. Interestingly, in our RNA-seq results (Table S1), most of the *HIST* transcripts were up regulated suggestive of aberrations in nucleosomes and DNA regulation possibly by ADNP mutations. Furthermore, *Hist1h3b* was downregulated in the *Adnp*-deficient mouse cerebral cortex and this was corrected by treatment with the ADNP snippet, drug candidate NAP^10^.

Further assessing RNA expression, *GRM3, MBP SNAP25* and *NGLN1* were all decreased in the cerebellum because of the ADNP mutation (Fig. 2, Fig. S2). These genes, directly associated with synapse plasticity and myelin function, have been implicated before with ADNP regulation in the *Adnp*^+/−^ mouse, with an increase in *Ngln2* in the male hippocampus and correction by NAP treatment^10^ with ADNP interacting with key regulatory proteins at the synapse^11^ and regulating the excitatory glutamategic synapse^29^. ADNP regulation of *MBP* is further directly linked to white matter lesions in the ADNP syndrome^6^.

Further leading from brain to peripheral tissues, we have previously shown a direct binding of ADNP to the *Myl2* promoter, the most downregulated gene in *Adnp* knockout mouse embryos^27^ and strongly affected by *Adnp* haploinsufficiency^7^. In normal tissues this gene is expressed mainly in the cardiac muscle and in smooth muscles and may be directly linked to the digestive problems, hypotonia and occasional cardiac problems noted in ADNP children^6^.

Taken together also in comparison to the in depth phenotypic analysis of 78 ADNP children^6^, our gene expression results correlate with the developmental delays measured in the ADNP children (100%), the cognitive, motor and gastrointestinal problems observed in the majority of the children, as well as the potential hormonal imbalance and urogenital abnormalities. Furthermore, our recent findings also associated somatic brain mutations in ADNP (among other genes) as possible risk factors/driving pathologies of Alzheimer’s disease (the most prevalent tauopathy). Importantly, a substantial overlap between autism/ID and Alzheimer’s brain mutations was discovered as well as a relatively high proportion of mutations in cytoskeletal genes^19^.

Finally, we have now extended our previous finding with truncating ADNP mutations (p.Tyr719* and p.Arg730*) reducing microtubule Tau interaction and microtubule dynamics rescued by NAP treatment^19^ to a shorter truncating mutation (p.Ser404*), still showing a significant protection by NAP treatment. Together these results corroborate ADNP/NAP microtubule-Tau protective mechanism, while offering potential clinical relief against tauopathy.

In terms of strength and limitations, our current study reports for the first time tauopathy in an ADNP syndrome child. We have utilized the only available postmortem tissue of the ADNP syndrome to-date and compared it to a young adult control brain. Gene expression analysis identified Tau (MAPT) as a central deregulated protein in the ADNP syndrome brain. NAP enhanced Tau—microtubule binding in the face of ADNP mutation, which is potentially protective against juvenile tauopathy. Study limitations reside in the constraints of a case report, comparison to a young adult and the focus on the ADNP syndrome. It should also be taken into consideration that previous brain MRI (unavailable) showed diffuse cortical atrophy of the brain parenchyma, marked reduction in volume of white matter as well as gliosis in both frontal and temporoparietal lobes that could indicate the sequelae of acute hepatic encephalopathy including a 3.3 cm large arachnoid cyst in middle cranial fossa. This pathology may have affected gene expression and tauopathy. Regardless, similar brain pathologies are shared by other ADNP patients with none (except for the current case) undergoing liver transplantations^6^. Furthermore, the ADNP syndrome shares similarities with other ASD/ID syndromes (https://www.orpha.net/consor/cgi-bin/OC_Exp.php?lng=EN&Expert=404448) broadening result impact. Lastly, a recent study in different ASD mouse models showing a strong Tau involvement may generalize our findings^37^. Specifically, Tau reduction by transgenic mouse mating (*Mapt*^*+/−*^ or *Mapt*^*−/−*^ compared with *Mapt*^*+/+*^controls) ameliorated autistic features (e.g., repetitive self-grooming and social disinterest) in two different mouse lines, *Scn1a*^*RX/+*^ (sodium voltage-gated channel alpha subunit 1A-R1407* modeling Dravet syndrome) and *Cntnap2*^*–/–*^ mice (contactin associated protein 2 representing a relatively prevalent autism-causing protein deficiency). Lowering tau also reduced megalencephaly toward normalization of brain size in these mice. In contrast, in a mouse line of *Shank3B*^*–/–*^ (SH3 and multiple ankyrin repeat domains 3 deficiency, modeling the Phelan McDermid Syndrome), Tau reduction was not beneficial. The authors suggested that the PI3K/Akt/mTOR (protease inhibitor 13/AKT Serine/Threonine kinase/mTOR) pathway, activated by interactions of Tau and PTEN (phosphatase and tensin homolog) plays a key role in the ASD development in the *Scn1a*^*RX/+*^ and the *Cntnap2*^*–/–*^ cases, but not in the *Shank3B*^*–/–*^ mouse^37^. Notably, MTOR was found to be regulated by ADNP in human postmortem tissues (Fig. 2) and in the *Adnp*^*+/−*^ mouse model^10^. Regardless of the precise mechanism/pathway that may vary among different autistic syndromes (e.g., the ADNP syndrome is not characterized by megalencephaly), Tau pathology emerges as a key player in versatile ASD syndromes.

In conclusion, the robust changes in gene expression linked to nervous tissue and organ development coupled to early onset brain tauopathy and exhibiting strong similarities to the *Adnp*-deficient inbred^15^ and outbred mouse^10^, places ADNP as a highly conserved master gene regulator of human development. The identification of taoupathy, which might be associated with the observed MRI pathology in the ADNP patients^6^ and potentially detected by advanced tau tangle imaging technologies^38^ coupled to the corrective effects of NAP (drug name CP201)^19^, paves the path for precision disease modifying therapeutics.

## Supplementary information

Supplementary Information

Supplementary Table S1

Supplementary Table S2

Supplementary Table S3

Supplementary Table S4

## References

[1] Bassan M (1999). Complete sequence of a novel protein containing a femtomolar-activity-dependent neuroprotective peptide. J. Neurochem..

[2] Zamostiano R (2001). Cloning and characterization of the human activity-dependent neuroprotective protein. J. Biol. Chem..

[3] Helsmoortel C (2014). A SWI/SNF-related autism syndrome caused by de novo mutations in ADNP. Nat. Genet..

[4] Deciphering Developmental Disorders S. (2017). Prevalence and architecture of de novo mutations in developmental disorders. Nature.

[5] Stessman HA (2017). Targeted sequencing identifies 91 neurodevelopmental-disorder risk genes with autism and developmental-disability biases. Nat. Genet..

[6] Van Dijck A (2019). Clinical presentation of a complex neurodevelopmental disorder caused by mutations in ADNP. Biol. Psychiatry.

[7] Amram N. et al. Sexual divergence in microtubule function: the novel intranasal microtubule targeting SKIP normalizes axonal transport and enhances memory. *Mol. Psychiatry* **21**, 1467–1476 (2016). 10.1038/mp.2015.208 26782054

[8] Merenlender-Wagner A (2015). Autophagy has a key role in the pathophysiology of schizophrenia. Mol. Psychiatry.

[9] Mandel S, Spivak-Pohis I, Gozes I (2008). ADNP differential nucleus/cytoplasm localization in neurons suggests multiple roles in neuronal differentiation and maintenance. J. Mol. Neurosci..

[10] Hacohen-Kleiman G (2018). Activity-dependent neuroprotective protein deficiency models synaptic and developmental phenotypes of autism-like syndrome. J. Clin. Investig..

[11] Oz S (2014). The NAP motif of activity-dependent neuroprotective protein (ADNP) regulates dendritic spines through microtubule end binding proteins. Mol. Psychiatry.

[12] Ivashko-Pachima Y, Sayas CL, Malishkevich A, Gozes I (2017). ADNP/NAP dramatically increase microtubule end-binding protein-Tau interaction: a novel avenue for protection against tauopathy. Mol. Psychiatry.

[13] Oz S, Ivashko-Pachima Y, Gozes I (2012). The ADNP derived peptide, NAP modulates the tubulin pool: implication for neurotrophic and neuroprotective activities. PLoS ONE.

[14] Jouroukhin Y (2013). NAP (davunetide) modifies disease progression in a mouse model of severe neurodegeneration: protection against impairments in axonal transport. Neurobiol. Dis..

[15] Vulih-Shultzman I (2007). Activity-dependent neuroprotective protein snippet NAP reduces tau hyperphosphorylation and enhances learning in a novel transgenic mouse model. J. Pharmacol. Exp. Ther..

[16] Quraishe S, Cowan CM, Mudher A (2013). NAP (davunetide) rescues neuronal dysfunction in a Drosophila model of tauopathy. Mol. Psychiatry.

[17] Ivashko-Pachima Y, Maor-Nof M, Gozes I (2019). NAP (davunetide) preferential interaction with dynamic 3-repeat Tau explains differential protection in selected tauopathies. PLoS ONE.

[18] Gozes I (2017). Premature primary tooth eruption in cognitive/motor-delayed ADNP-mutated children. Transl. Psychiatry.

[19] Ivashko-Pachima Y. et al. Discovery of autism/intellectual disability somatic mutations in Alzheimer’s brains: mutated ADNP cytoskeletal impairments and repair as a case study. *Mol. Psychiatry* (2019). (EPUB) 10.1038/s41380-019-0563-5 PMC8159740 31664177

[20] Carithers LJ (2015). A novel approach to high-quality postmortem tissue procurement: the GTEx project. Biopreserv. Biobank..

[21] Subramanian A (2005). Gene set enrichment analysis: a knowledge-based approach for interpreting genome-wide expression profiles. Proc. Natl Acad. Sci. USA.

[22] Hait TA (2019). The EXPANDER integrated platform for transcriptome analysis. J. Mol. Biol..

[23] Kanehisa M, Goto S (2000). KEGG: kyoto encyclopedia of genes and genomes. Nucleic Acids Res..

[24] Liberzon A (2015). The Molecular Signatures Database (MSigDB) hallmark gene set collection. Cell Syst..

[25] Escher U (2018). Anti-inflammatory effects of the octapeptide NAP in human microbiota-associated mice suffering from Subacute Ileitis. Eur. J. Microbiol. Immunol..

[26] Heimesaat MM, Giladi E, Kuhl AA, Bereswill S, Gozes I (2018). The octapetide NAP alleviates intestinal and extra-intestinal anti-inflammatory sequelae of acute experimental colitis. Peptides.

[27] Mandel S, Rechavi G, Gozes I (2007). Activity-dependent neuroprotective protein (ADNP) differentially interacts with chromatin to regulate genes essential for embryogenesis. Dev. Biol..

[28] Yeung J, Ha TJ, Swanson DJ, Goldowitz D (2016). A novel and multivalent role of Pax6 in cerebellar development.. J. Neurosci..

[29] Sragovich S (2019). The autism/neuroprotection-linked ADNP/NAP regulate the excitatory glutamatergic synapse. Transl. Psychiatry.

[30] Glaser T (1994). PAX6 gene dosage effect in a family with congenital cataracts, aniridia, anophthalmia and central nervous system defects. Nat. Genet..

[31] Heins N (2002). Glial cells generate neurons: the role of the transcription factor Pax6. Nat. Neurosci..

[32] Mandel S, Gozes I (2007). Activity-dependent neuroprotective protein constitutes a novel element in the SWI/SNF chromatin remodeling complex. J. Biol. Chem..

[33] Hacohen-Kleiman G (2019). Atypical auditory brainstem response and protein expression aberrations related to ASD and hearing loss in the adnp haploinsufficient mouse brain. Neurochem. Res..

[34] Muenke M (1994). A common mutation in the fibroblast growth factor receptor 1 gene in Pfeiffer syndrome. Nat. Genet..

[35] Villanueva C, de Roux N (2010). FGFR1 mutations in Kallmann syndrome. Front. Horm. Res..

[36] Trokovic N, Trokovic R, Mai P, Partanen J (2003). Fgfr1 regulates patterning of the pharyngeal region. Genes Dev..

[37] Tai C (2020). Tau reduction prevents key features of autism in mouse models. Neuorn.

[38] La Joie R. et al. Prospective longitudinal atrophy in Alzheimer’s disease correlates with the intensity and topography of baseline tau-PET. *Sci. Transl. Med.* **12**, eaau5732 (2020). 10.1126/scitranslmed.aau5732 PMC7035952 31894103

[39] Tomasoni R (2013). SNAP-25 regulates spine formation through postsynaptic binding to p140Cap. Nat. Commun..

[40] Malishkevich A, Leyk J, Goldbaum O, Richter-Landsberg C, Gozes I (2015). ADNP/ADNP2 expression in oligodendrocytes: implication for myelin-related neurodevelopment. J. Mol. Neurosci..

[41] Malishkevich A (2015). Activity-dependent neuroprotective protein (ADNP) exhibits striking sexual dichotomy impacting on autistic and Alzheimer’s pathologies. Transl. Psychiatry.

[42] Pinhasov A (2003). Activity-dependent neuroprotective protein: a novel gene essential for brain formation. Dev. Brain Res..

[43] Vaisburd S, Shemer Z, Yeheskel A, Giladi E, Gozes I (2015). Risperidone and NAP protect cognition and normalize gene expression in a schizophrenia mouse model. Sci. Rep..

