# Supplementary material for: Tauopathy in the young autistic brain: novel biomarker and therapeutic target
Source: Transl Psychiatry. 2020 Jul 13;10:228. doi: 10.1038/s41398-020-00904-4 (PMC7359319; doi:10.1038/s41398-020-00904-4)
Supplement: Supplementary file 1 — Supplementary Information [file 41398_2020_904_MOESM1_ESM.pdf]

Supplementary Methods and Results

Tauopathy in the Young Autistic Brain: Novel Biomarker and Therapeutic Target

Iris Grigg[1], Yanina Ivashko-Pachima[1]*, Tom Aharon Hait[1,2]*, Vlasta Korenková[3], Olga Touloumi[4], Roza Lagoudaki[4], Anke Van Dijck[5], Zlatko Marusic[6], Mirna Anicic[7], Jurica Vukovic[7], R. Frank Kooy[5], Nikolaos Grigoriadis[4], Illana Gozes[1]**

[1]Elton Laboratory for Neuroendocrinology, Department of Human Molecular Genetics and Biochemistry, Sackler Faculty of Medicine, Sagol School of Neuroscience and Adams Super Center for Brain Studies, Tel Aviv University, Tel Aviv, Israel.

[2]The Blavatnik School of Computer Science, Tel Aviv University, Tel Aviv, Israel

[3]BIOCEV, Institute of Biotechnology CAS, Průmyslová 595, 252 50 Vestec, Czech Republic.

[4]Department of Neurology, Laboratory of Experimental Neurology, AHEPA University Hospital, Aristotle University of Thessaloniki, Thessaloniki, Greece.

[5]Department of Medical Genetics, University of Antwerp, Antwerp, Belgium.

[6]Clinical Department of Pathology and Cytology, University Hospital Centre Zagreb, Zagreb, Croatia.

University Hospital Centre Zagreb

[7]Department of Pediatrics, Division of Pediatric Gastroenterology, Hepatology and Nutrition

***Corresponding author:**

E-mail: igozes@tauex.tau.ac.il

Supplemental Methods

Immunohistochemistry

Paraffin sections were deparaffinized and hydrated in xylene and alcohol solutions, rinsed with TBS. Citrate buffer was used for antigen retrieval and the endogenous hyperoxidase was blocked with 3% H_2O_2 in methanol. After incubation in blocking buffer (FBS 10%), the sections were further incubated (overnight) with primary antibodies against phosphorylated Tau [AT8 (MN1020, Thermo Scientific), AT180 (MN1040, Thermo Scientific)], PSD95(ab12093, Abcam), NMDAR1(ab193310, Abcam), Glut1 (ab31232, Abcam), MAP2 (MAB33418, Millipore). The secondary antibodies used were goat anti mouse (sc2039, Santa Cruz), goat anti rabbit (BA1000, Vector) and rabbit anti goat (AP106B, Millipore), immunoreactions were visualized using 3,3′-Diaminobenzidine (DAB) as chromogen. Counterstaining was performed with hematoxyline to show nuclear staining[1]. Double immunofluorescence was performed for PSD95/NMDR. Goat anti-mouse IgG (20010, Biotium, CF 488A) and donkey anti-goat IgG (20039, Biotium, CF 555), were used as secondary antibodies. Slides were mounted with Dapi (23004, Biotium).

Neuronal-like cell models[2]

Mouse neuroblastoma N1E-115 cells (a kind gift of Dr. Laura Sayas)[3] were maintained in Dulbecco's modified Eagle's medium, 10% fetal bovine serum, 2 mM glutamine and 100 U ml − 1 penicillin, 100 mg ml− 1 streptomycin (Biological Industries, Beit Haemek, Israel). The cells were incubated in 95% air/5% CO2 in a humidified incubator at 37 °C.

Cell differentiation and co-transfection of overexpression plasmids[2]

Cultured N1E-115 cells were plated on 35 mm dishes (81156, 60 μ-Dish, Ibidi, Martinsried, Germany) at a concentration of $1.25*10^4$ cells per dish and then were differentiated with reduced fetal bovine serum (2%) and DMSO (1.25%) containing medium for 5 days before transfection. 48hrs before experiments N1E-115 cells were co-transfected with 1μg mCherry-Tau plasmid with or without 2μg of plasmids, coding to GFP conjugated to full-length human ADNP, or ADNP carried mutations (p.Ser404*). 1:3 ratios between total amount of transfected DNA and transfection reagent were used according to manufacture guidance (Lipofectamine2000, Thermo Fisher Scientific, Waltham, MA, USA) in all subsequent experiments.

All cell lines have been tested for mycoplasma contamination and proven to be clean. The kit used was EZ-PCR mycoplasma detection kit (Biological Industries, Beit Haemek, Israel).

Plasmid construction

Protein expressing plasmids were constructed as previously described[2] based on pEGFP-C1 backbone and express full-length ADNP or the truncated form of p.Ser404* of ADNP proteins (Supplemental Fig. S4A).

Inserts of cDNA carrying unique mutations were obtained from mRNA extracted from patient-derived lymphoblastoid cell lines and cDNA with full-length human ADNP was obtained from a control lymphoblastoid cell line with no mutation[4]. Protein expressions were verified by fluorescent imaging and immunoblotting analysis.

Fluorescence recovery after photobleaching (FRAP)[2]

Differentiated N1E-115 cells were transfected with mCherry-Tau with or without GFP-conjugated to full-length ADNP or p.Ser404* ADNP, and imaged 48hrs after transfection. An ROI for photobleaching was drowned in the proximal cell branches. mCherry-Tau was bleached with a 587nm argon laser, and fluorescence recovery was collected within wavelengths of 610-650nm. 80 images were taken every 0.74sec immediately after bleaching. After FRAP imaging all samples were also treated with NAP in final concentrations of 10^{-12}M, and after 4hrs time-lapse imaging was done again under the same conditions. Fluorescence signals were quantified with Fiji[5], obtained data were normalized with easyFRAP[6,7] and FRAP recovery curves were fitted by a one-phase exponential association function by GraphPad Prism6 (GraphPad Software, Inc., La Jolla, CA, USA) while samples with $R^2<0.9$ were excluded.

Lymphoblastoids[8] and library of ADNP mutated cell-lines, derived from ASD patients

A representative LCL from healthy adult donors was obtained from Dr. David Gurwitz at the National Laboratory for the Genetics of Israeli Populations (NLGIP; http://nlgip.tau.ac.il), Tel Aviv University. Two ADNP-mutated LCLs were purchased from the Simon Simplex Collection, SSC04121=ADNP (protein) p.Lys408Valfs*31 and SSC08311= p.Tyr719* and one was generated from peripheral blood lymphocytes donated by consenting patient and guardians/physicians (Professor Orly Elpeleg, Hadassah, Hebrew University Medical Center, Jerusalem)[8]. For details see Table S1. Lymphoblastoid cells were grown in an RPMI-1640 medium supplemented with 10% fetal bovine serum (FBS), 1% L- Glutamate and 1% Penicillin – Streptomycin (P/S) mixture. All materials were purchased from Biological Industries Ltd. Kibbutz Beit Haemek, 25115, Israel. Cells were grown at 37°C, 8% CO_2 and 99% humidity.

Selected genes for qPCR microarray analysis

Table 1, prepared based on our RNA-seq data (GEO, GSE81268)[4,9] lists the genes that showed the highest change when comparing ADNP mutated lymphoblastoid cells to a control lymphoblastoid cell line. Table 2 lists additional genes of interest that changed either as consequence of *Adnp* deficiency or as a results of NAP (CP201) treatment or of both.

qPCR microarray analysis

Before performing BioMark analysis the samples were pre-amplified. The pre-amplification reaction was done as follows: 2µl of cDNA (10ng RNA/µl) was mixed with 1.2µl of 208nM primer mix (Table S4 - Online Resource 6; all primers were mixed together, final concentration of each primer 25nM), 5µl of iQ Supermix (BioRad, Prague, Czech Republic) and 1.8µl of H_2O. The mixture was first incubated for 10 min at 95°C, followed by 18 cycles of 15 sec at 95°C, and finally 4 min at 59°C. Pre-amplified cDNA was diluted ×20. The real-time PCR reactions were carried out in GE Dynamic array 48×48 in a BioMark HD System (Fluidigm, San Francisco, California). 5µl of Fluidigm sample premix consisted of 1µl of ×20 diluted pre-amplified cDNA, 0.25µl of ×20 DNA Binding Dye Sample Loading Reagent (Fluidigm), 2.5µl of SsoFast™ EvaGreen® Supermix (Bio-Rad, Czech Republic), 0.1µl of ×4 diluted ROX (Invitrogen, USA) and 1.15µl of RNase/DNase-free water. Each 5µl assay premix consisted of 2.5µl of 10µM primers (forward and reversed at a final concentration of 500nM) and 2.5µl of DA Assay Loading Reagent (Fluidigm, USA). Thermal conditions for qPCR were: 98°C for 40 sec, 40 cycles of 95°C for 10 sec, and 60°C for 40 sec. The PPIA (Peptidylprolyl isomerase A) and PGK1 (Phosphoglycerate kinase 1) reference genes were selected from several reference gene candidates by Normfinder (GenEx Enterprise, MultiD Analyses, Sweden). The data were collected using BioMark 3.1.2 Data Collection software and analyzed by BioMark Real-Time PCR Analysis Software 3.1.3. (Fludigm, USA).

The cut-off value for Cq was set at 25 and values higher than that were replaced by the Cq value of 25. The missing data were filled with maximum of a column plus 1. Data were normalized with PPIA and PGK1. The fold change in expression was calculated using the $2^{-\Delta\Delta Cq}$ method[10] for each sample and then expressed as the mean of all these fold changes. The control was set at 100% and experimental samples were compared to the control.

Data Availability

All data are available either in the current paper or in online resources as outlined above and below.

Supplemental Results

List of Figures:

Fig. S1- **Patient mutation validation**

Fig. S2- **Heatmaps of relative expression between mutated ADNP child and healthy samples**

Fig. S3- **Distributions of ADNP syndrome, HPA, and GTEx expression values**

Fig. S4- **The ADNP syndrome olfactory bulb and hypothalamus exhibit intensive tauopathy, not detected in the corpus callosum and the trigeminal nerve**

Fig. S5A, B- **Plasmid maps and verification of plasmid expression**

Fig. S6A, 6B- **Immunohistochemistry results of PSD95 and NMDAR1 in the hippocampal area (case and control)**

Fig. S7- **Spearman correlation test between datasets**

Figure S1

Patient mutation validation

Figure S1. DNA extracted from the kidney, was Sanger sequenced at Hylabs (Rehovot, Israel). Using the ADNP primers:

FWD: 5'- TGAAACTATGTTCCAACTTCCTG - 3'   REV: 5' - CAGTATTCCAACCCAATGTCA - 3'

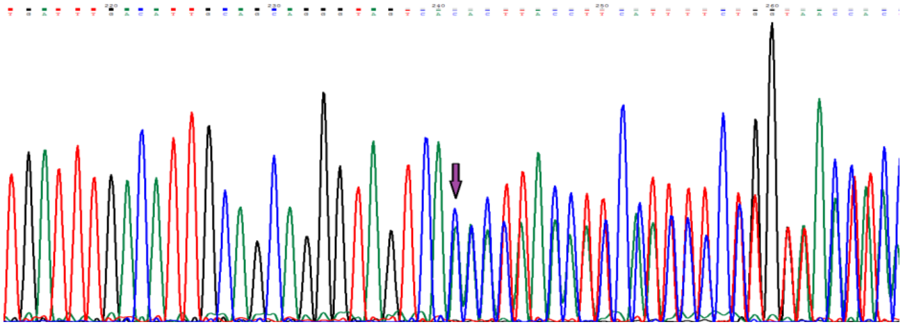

Figure S2

Heatmaps of relative expression between mutated ADNP child and healthy samples

Each heatmap presents the standardized expression of a single gene out of 38 genes selected for the analysis (Table 1 & 2; Table S3; Methods). We also added a heatmap for TBP housekeeping gene to make sure that the expression values in the healthy samples are valid. Values underlined within each cell. Black cells with 'NA' (not available) values denote missing data due to missing tissues or expression values in HPA/GTEx datasets.

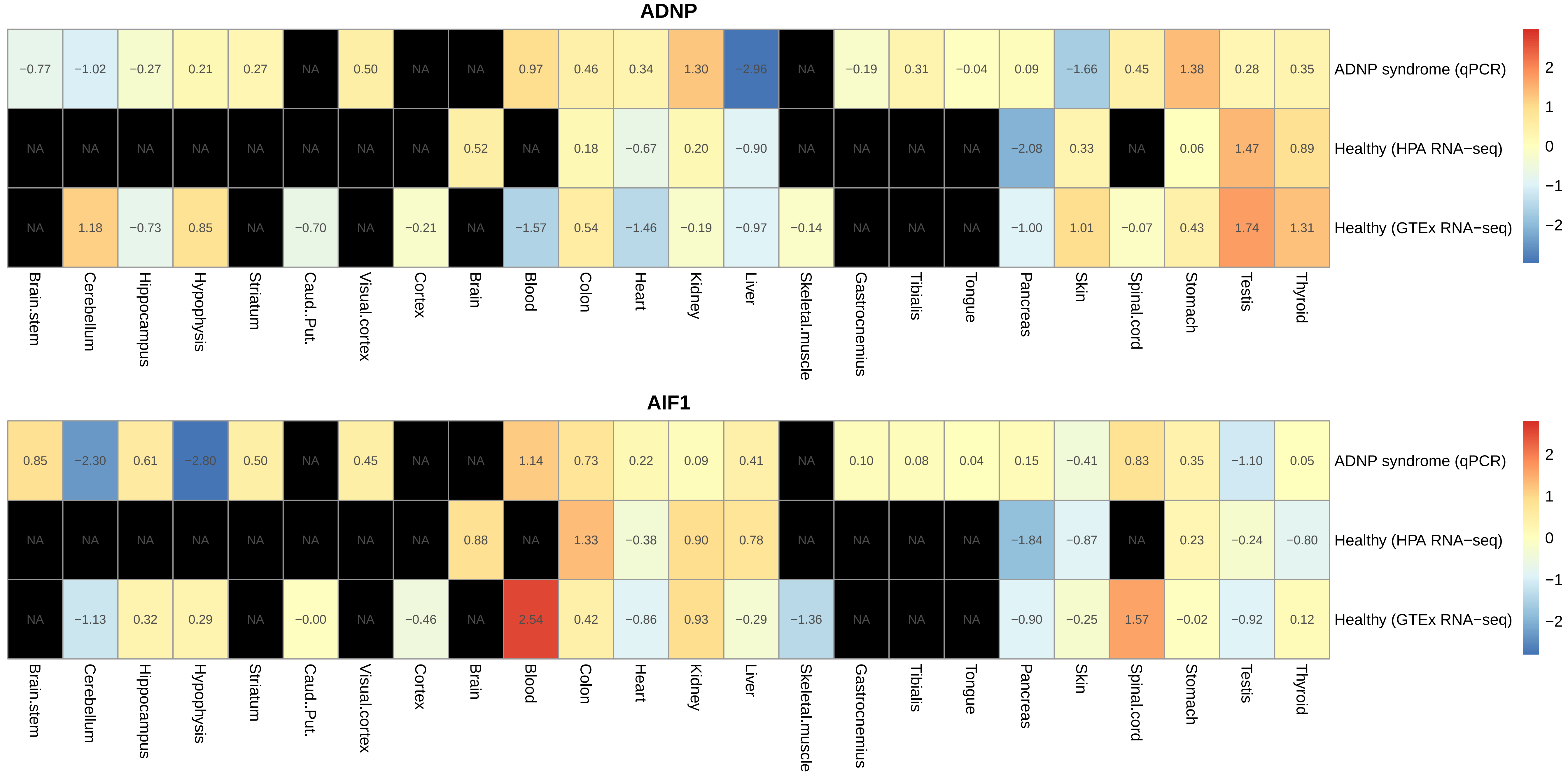

ADNP
ADNP syndrome (qPCR)
−0.77 −1.02 −0.27 0.21 0.27 NA 0.50 NA NA 0.97 0.46 0.34 1.30 −2.96 NA −0.19 0.31 −0.04 0.09 −1.66 0.45 1.38 0.28 0.35
Healthy (HPA RNA−seq)
NA NA NA NA NA NA NA NA 0.52 NA 0.18 −0.67 0.20 −0.90 NA NA NA NA −2.08 0.33 NA 0.06 1.47 0.89
Healthy (GTEx RNA−seq)
NA 1.18 −0.73 0.85 NA −0.70 NA −0.21 NA −1.57 0.54 −1.46 −0.19 −0.97 −0.14 NA NA NA −1.00 1.01 −0.07 0.43 1.74 1.31
Brain.stem
Cerebellum
Hippocampus
Hypophysis
Striatum
Caud..Put.
Visual.cortex
Cortex
Brain
Blood
Colon
Heart
Kidney
Liver
Skeletal.muscle
Gastrocnemius
Tibialis
Tongue
Pancreas
Skin
Spinal.cord
Stomach
Testis
Thyroid
2
1
0
−1
−2
AIF1
ADNP syndrome (qPCR)
0.85 −2.30 0.61 −2.80 0.50 NA 0.45 NA NA 1.14 0.73 0.22 0.09 0.41 NA 0.10 0.08 0.04 0.15 −0.41 0.83 0.35 −1.10 0.05
Healthy (HPA RNA−seq)
NA NA NA NA NA NA NA NA 0.88 NA 1.33 −0.38 0.90 0.78 NA NA NA NA −1.84 −0.87 NA 0.23 −0.24 −0.80
Healthy (GTEx RNA−seq)
NA −1.13 0.32 0.29 NA −0.00 NA −0.46 NA 2.54 0.42 −0.86 0.93 −0.29 −1.36 NA NA NA −0.90 −0.25 1.57 −0.02 −0.92 0.12
Brain.stem
Cerebellum
Hippocampus
Hypophysis
Striatum
Caud..Put.
Visual.cortex
Cortex
Brain
Blood
Colon
Heart
Kidney
Liver
Skeletal.muscle
Gastrocnemius
Tibialis
Tongue
Pancreas
Skin
Spinal.cord
Stomach
Testis
Thyroid
2
1
0
−1
−2

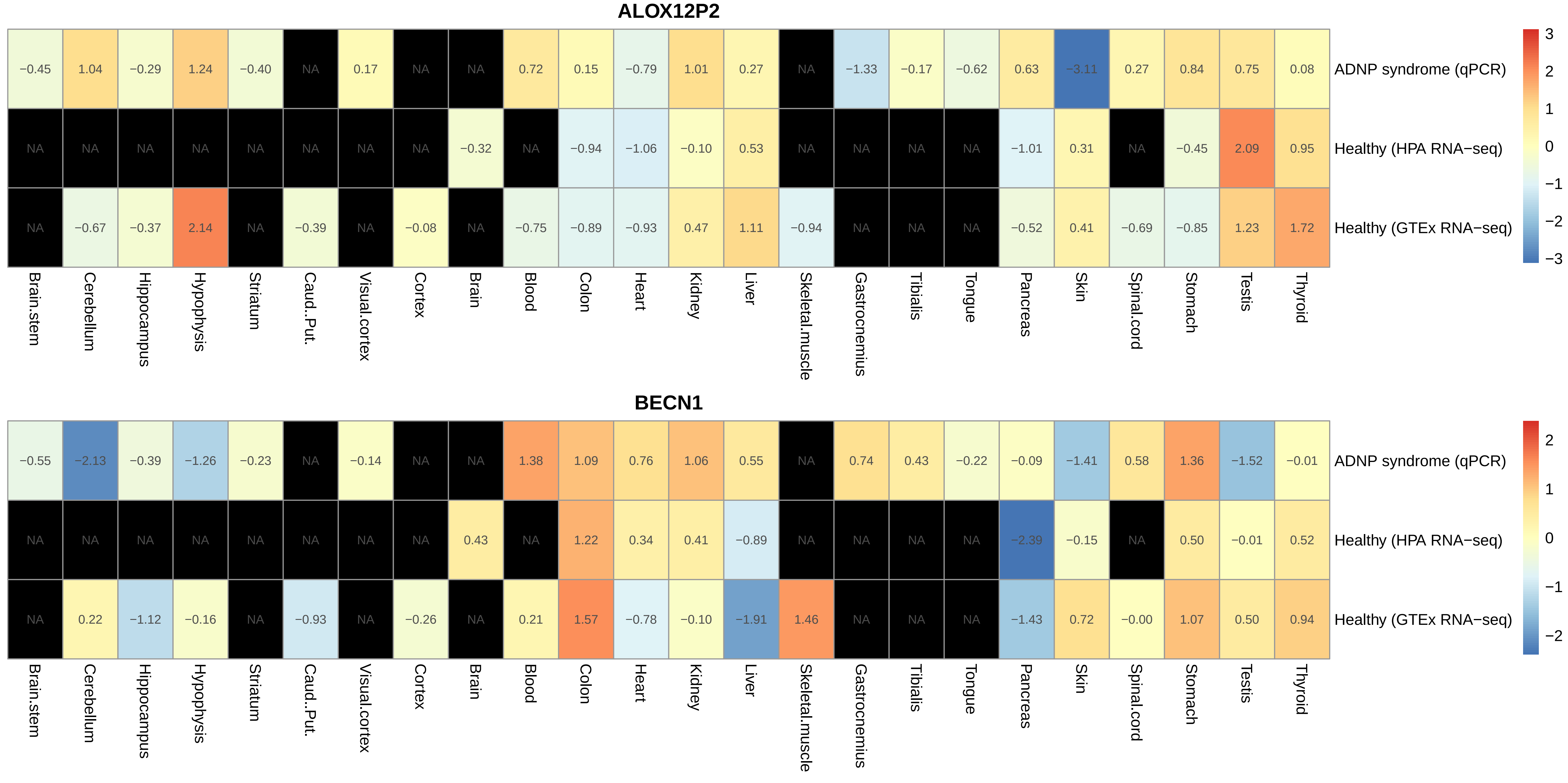
ALOX12P2
ADNP syndrome (qPCR)
−0.45 1.04 −0.29 1.24 −0.40 NA 0.17 NA NA 0.72 0.15 −0.79 1.01 0.27 NA −1.33 −0.17 −0.62 0.63 −3.11 0.27 0.84 0.75 0.08
Healthy (HPA RNA−seq)
NA NA NA NA NA NA NA NA −0.32 NA −0.94 −1.06 −0.10 0.53 NA NA NA NA −1.01 0.31 NA −0.45 2.09 0.95
Healthy (GTEx RNA−seq)
NA −0.67 −0.37 2.14 NA −0.39 NA −0.08 NA −0.75 −0.89 −0.93 0.47 1.11 −0.94 NA NA NA −0.52 0.41 −0.69 −0.85 1.23 1.72
Brain.stem
Cerebellum
Hippocampus
Hypophysis
Striatum
Caud..Put.
Visual.cortex
Cortex
Brain
Blood
Colon
Heart
Kidney
Liver
Skeletal.muscle
Gastrocnemius
Tibialis
Tongue
Pancreas
Skin
Spinal.cord
Stomach
Testis
Thyroid
3
2
1
0
−1
−2
−3
BECN1
ADNP syndrome (qPCR)
−0.55 −2.13 −0.39 −1.26 −0.23 NA −0.14 NA NA 1.38 1.09 0.76 1.06 0.55 NA 0.74 0.43 −0.22 −0.09 −1.41 0.58 1.36 −1.52 −0.01
Healthy (HPA RNA−seq)
NA NA NA NA NA NA NA NA 0.43 NA 1.22 0.34 0.41 −0.89 NA NA NA NA −2.39 −0.15 NA 0.50 −0.01 0.52
Healthy (GTEx RNA−seq)
NA 0.22 −1.12 −0.16 NA −0.93 NA −0.26 NA 0.21 1.57 −0.78 −0.10 −1.91 1.46 NA NA NA −1.43 0.72 −0.00 1.07 0.50 0.94
Brain.stem
Cerebellum
Hippocampus
Hypophysis
Striatum
Caud..Put.
Visual.cortex
Cortex
Brain
Blood
Colon
Heart
Kidney
Liver
Skeletal.muscle
Gastrocnemius
Tibialis
Tongue
Pancreas
Skin
Spinal.cord
Stomach
Testis
Thyroid
2
1
0
−1
−2

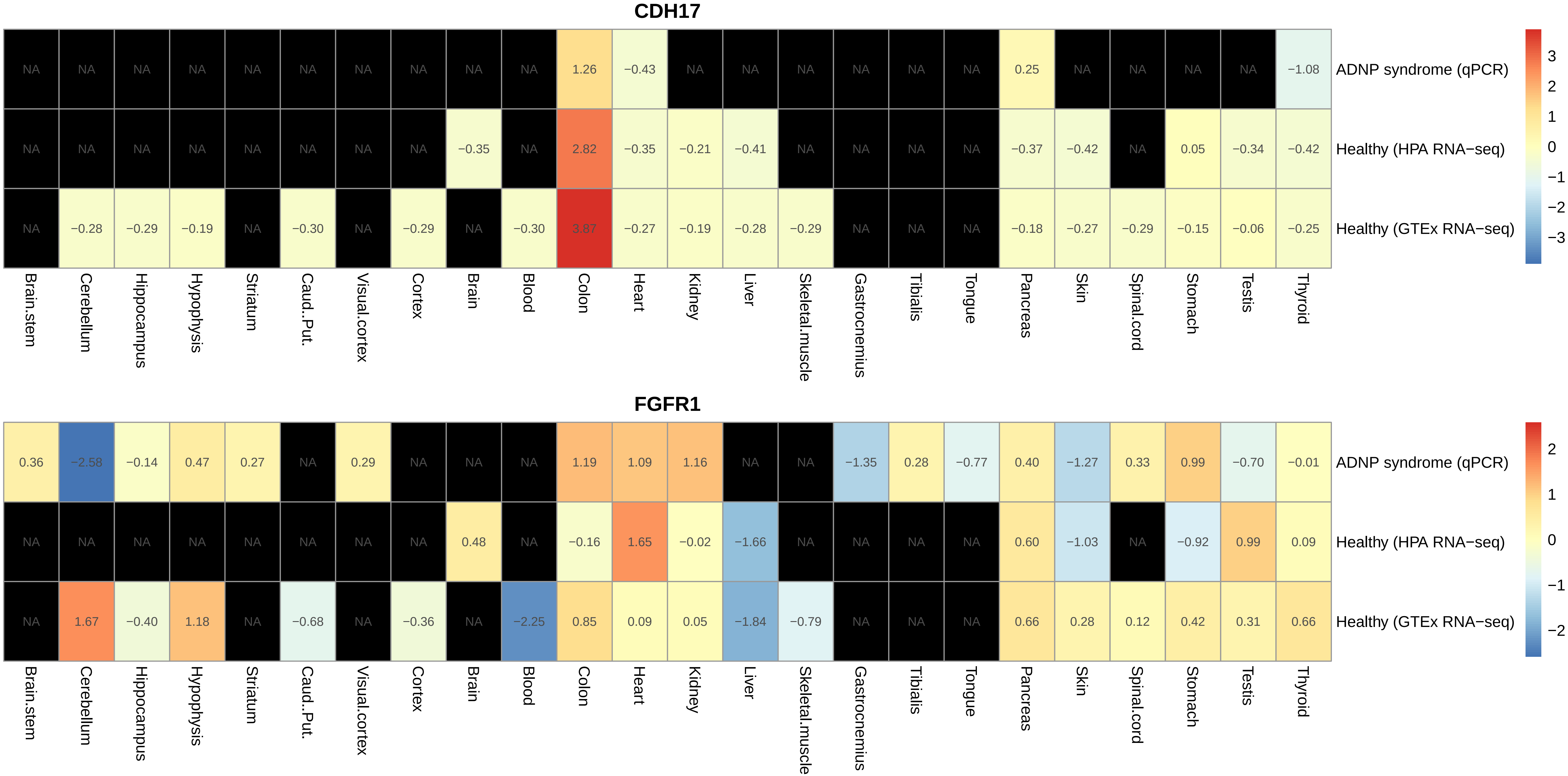
CDH17
FGFR1
ADNP syndrome (qPCR)
Healthy (HPA RNA−seq)
Healthy (GTEx RNA−seq)
Brain.stem
Cerebellum
Hippocampus
Hypophysis
Striatum
Caud..Put.
Visual.cortex
Cortex
Brain
Blood
Colon
Heart
Kidney
Liver
Skeletal.muscle
Gastrocnemius
Tibialis
Tongue
Pancreas
Skin
Spinal.cord
Stomach
Testis
Thyroid

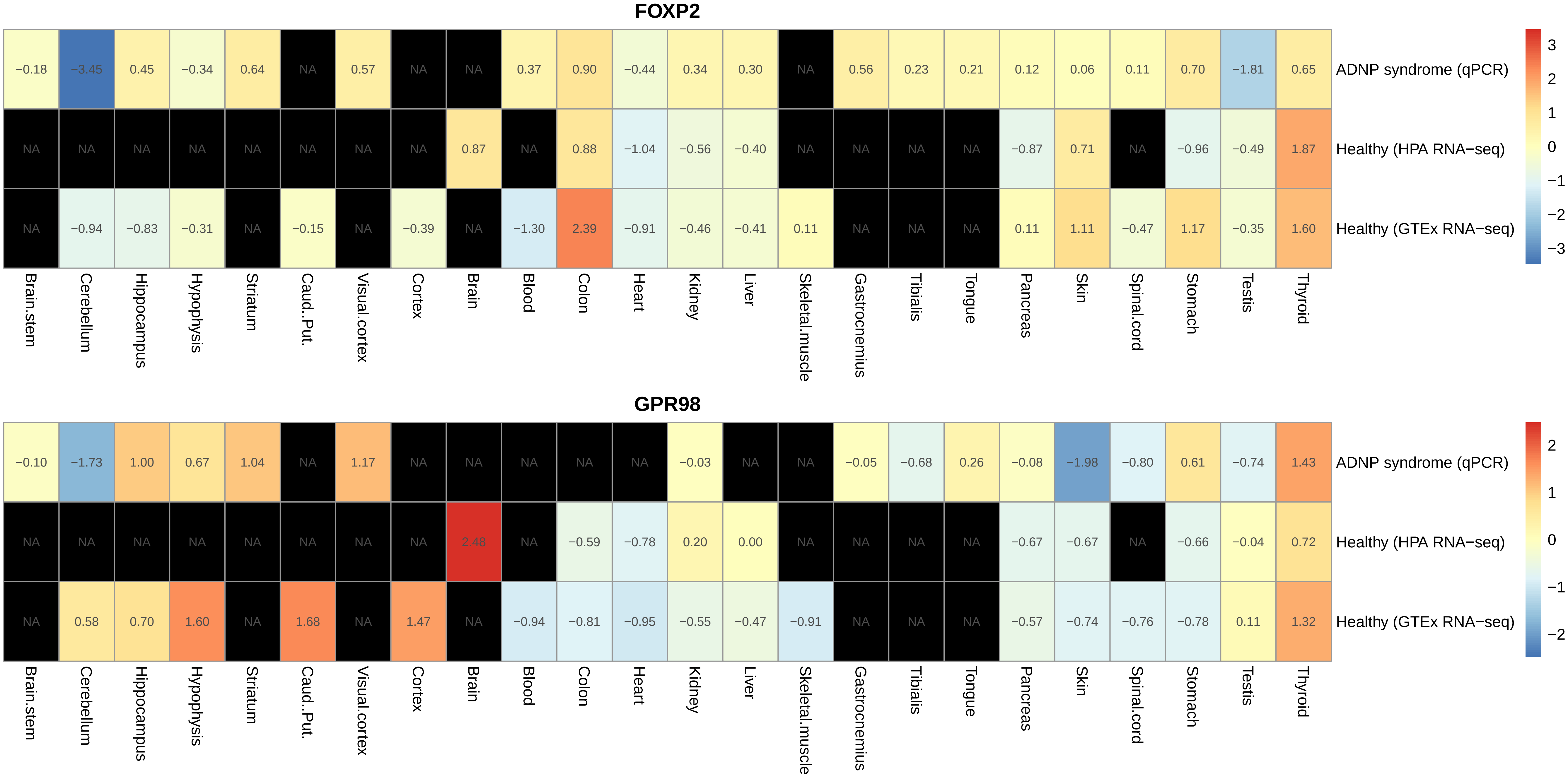
FOXP2
ADNP syndrome (qPCR)
Healthy (HPA RNA−seq)
Healthy (GTEx RNA−seq)
Brain.stem
Cerebellum
Hippocampus
Hypophysis
Striatum
Caud..Put.
Visual.cortex
Cortex
Brain
Blood
Colon
Heart
Kidney
Liver
Skeletal.muscle
Gastrocnemius
Tibialis
Tongue
Pancreas
Skin
Spinal.cord
Stomach
Testis
Thyroid
−0.18 −3.45 0.45 −0.34 0.64 NA 0.57 NA NA 0.37 0.90 −0.44 0.34 0.30 NA 0.56 0.23 0.21 0.12 0.06 0.11 0.70 −1.81 0.65
NA NA NA NA NA NA NA NA 0.87 NA 0.88 −1.04 −0.56 −0.40 NA NA NA NA −0.87 0.71 NA −0.96 −0.49 1.87
NA −0.94 −0.83 −0.31 NA −0.15 NA −0.39 NA −1.30 2.39 −0.91 −0.46 −0.41 0.11 NA NA NA 0.11 1.11 −0.47 1.17 −0.35 1.60
3
2
1
0
−1
−2
−3
GPR98
ADNP syndrome (qPCR)
Healthy (HPA RNA−seq)
Healthy (GTEx RNA−seq)
−0.10 −1.73 1.00 0.67 1.04 NA 1.17 NA NA NA NA NA −0.03 NA NA −0.05 −0.68 0.26 −0.08 −1.98 −0.80 0.61 −0.74 1.43
NA NA NA NA NA NA NA NA 2.48 NA −0.59 −0.78 0.20 0.00 NA NA NA NA −0.67 −0.67 NA −0.66 −0.04 0.72
NA 0.58 0.70 1.60 NA 1.68 NA 1.47 NA −0.94 −0.81 −0.95 −0.55 −0.47 −0.91 NA NA NA −0.57 −0.74 −0.76 −0.78 0.11 1.32
2
1
0
−1
−2

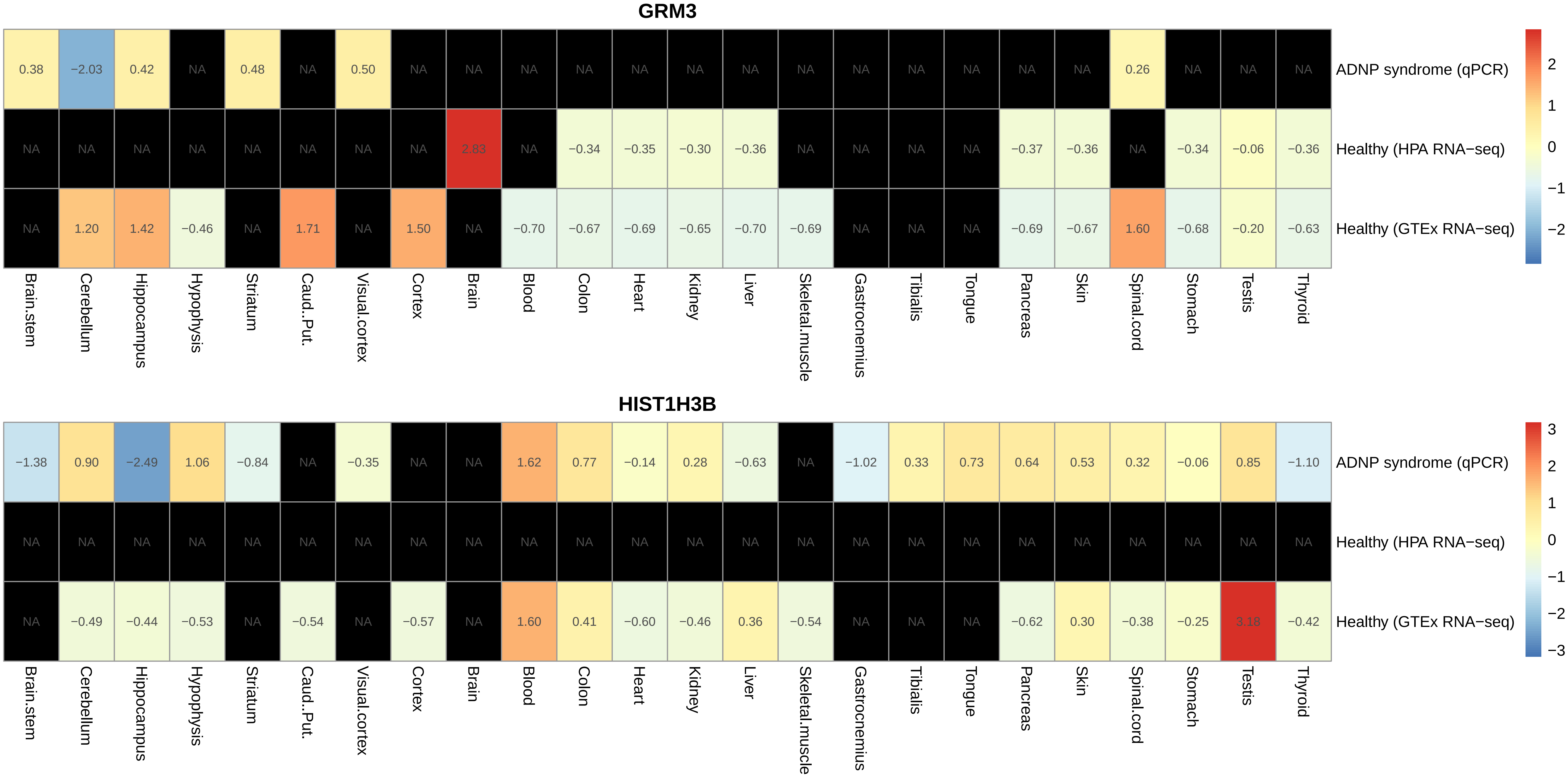
GRM3
ADNP syndrome (qPCR)
Healthy (HPA RNA−seq)
Healthy (GTEx RNA−seq)
Brain.stem
Cerebellum
Hippocampus
Hypophysis
Striatum
Caud..Put.
Visual.cortex
Cortex
Brain
Blood
Colon
Heart
Kidney
Liver
Skeletal.muscle
Gastrocnemius
Tibialis
Tongue
Pancreas
Skin
Spinal.cord
Stomach
Testis
Thyroid
HIST1H3B
ADNP syndrome (qPCR)
Healthy (HPA RNA−seq)
Healthy (GTEx RNA−seq)

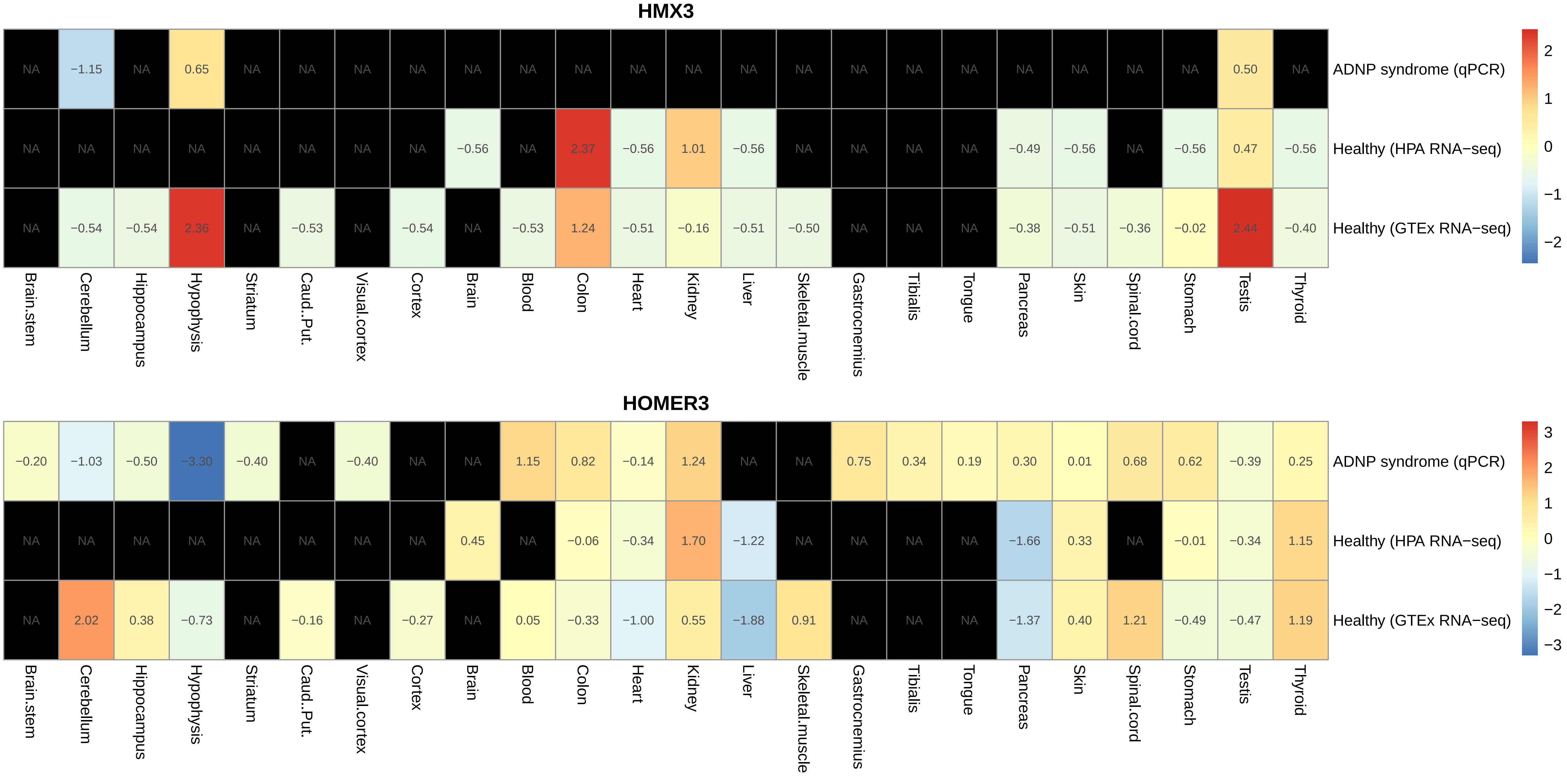
HMX3
NA −1.15 NA 0.65 NA NA NA NA NA NA NA NA NA NA NA NA NA NA NA NA NA NA 0.50 NA
ADNP syndrome (qPCR)
NA NA NA NA NA NA NA NA −0.56 NA 2.37 −0.56 1.01 −0.56 NA NA NA NA −0.49 −0.56 NA −0.56 0.47 −0.56
Healthy (HPA RNA−seq)
NA −0.54 −0.54 2.36 NA −0.53 NA −0.54 NA −0.53 1.24 −0.51 −0.16 −0.51 −0.50 NA NA NA −0.38 −0.51 −0.36 −0.02 2.44 −0.40
Healthy (GTEx RNA−seq)
2
1
0
−1
−2
Brain.stem
Cerebellum
Hippocampus
Hypophysis
Striatum
Caud..Put.
Visual.cortex
Cortex
Brain
Blood
Colon
Heart
Kidney
Liver
Skeletal.muscle
Gastrocnemius
Tibialis
Tongue
Pancreas
Skin
Spinal.cord
Stomach
Testis
Thyroid
HOMER3
−0.20 −1.03 −0.50 −3.30 −0.40 NA −0.40 NA NA 1.15 0.82 −0.14 1.24 NA NA 0.75 0.34 0.19 0.30 0.01 0.68 0.62 −0.39 0.25
ADNP syndrome (qPCR)
NA NA NA NA NA NA NA NA 0.45 NA −0.06 −0.34 1.70 −1.22 NA NA NA NA −1.66 0.33 NA −0.01 −0.34 1.15
Healthy (HPA RNA−seq)
NA 2.02 0.38 −0.73 NA −0.16 NA −0.27 NA 0.05 −0.33 −1.00 0.55 −1.88 0.91 NA NA NA −1.37 0.40 1.21 −0.49 −0.47 1.19
Healthy (GTEx RNA−seq)
3
2
1
0
−1
−2
−3
Brain.stem
Cerebellum
Hippocampus
Hypophysis
Striatum
Caud..Put.
Visual.cortex
Cortex
Brain
Blood
Colon
Heart
Kidney
Liver
Skeletal.muscle
Gastrocnemius
Tibialis
Tongue
Pancreas
Skin
Spinal.cord
Stomach
Testis
Thyroid

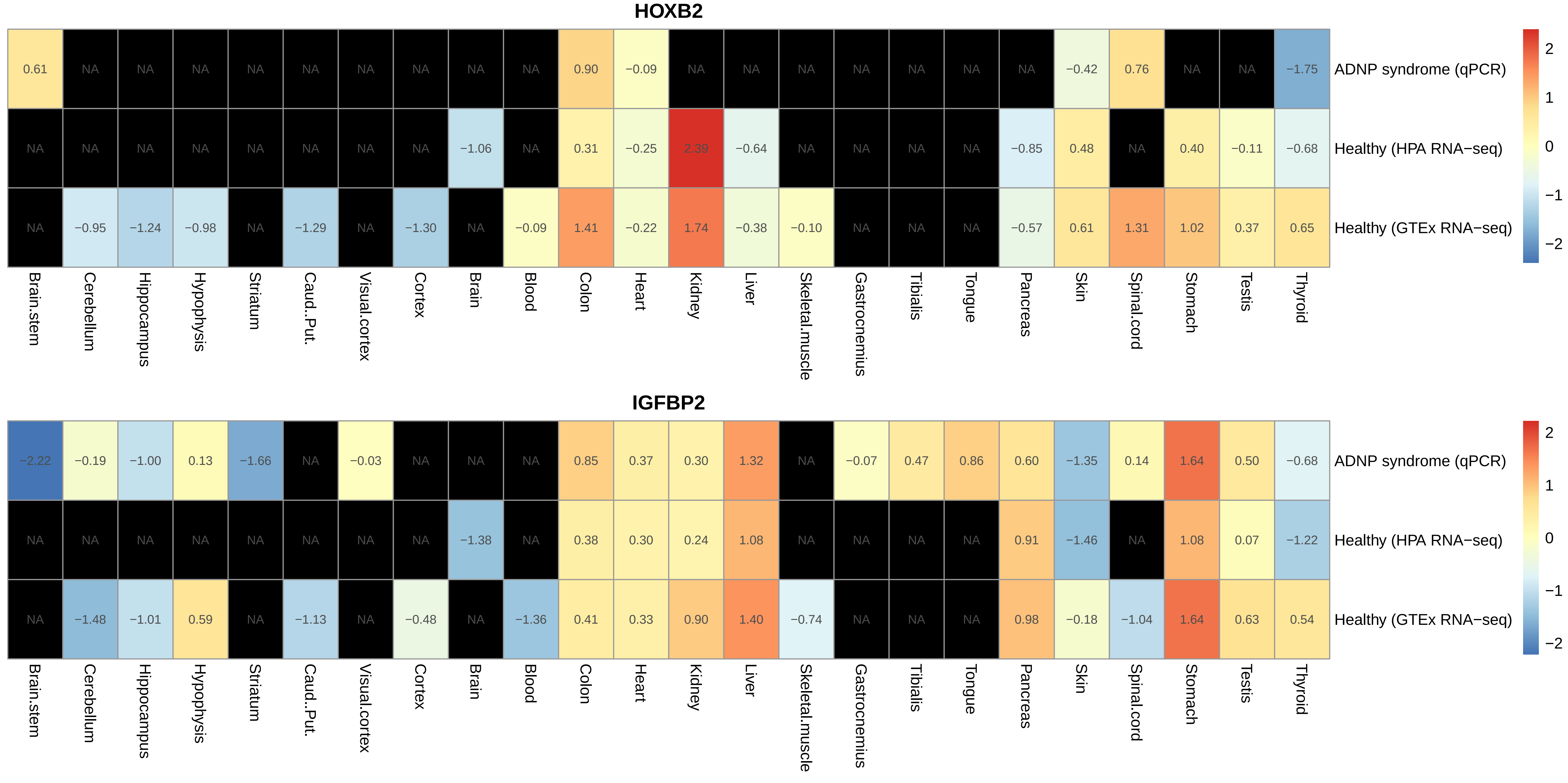
HOXB2
IGFBP2
ADNP syndrome (qPCR)
Healthy (HPA RNA−seq)
Healthy (GTEx RNA−seq)
Brain.stem
Cerebellum
Hippocampus
Hypophysis
Striatum
Caud..Put.
Visual.cortex
Cortex
Brain
Blood
Colon
Heart
Kidney
Liver
Skeletal.muscle
Gastrocnemius
Tibialis
Tongue
Pancreas
Skin
Spinal.cord
Stomach
Testis
Thyroid

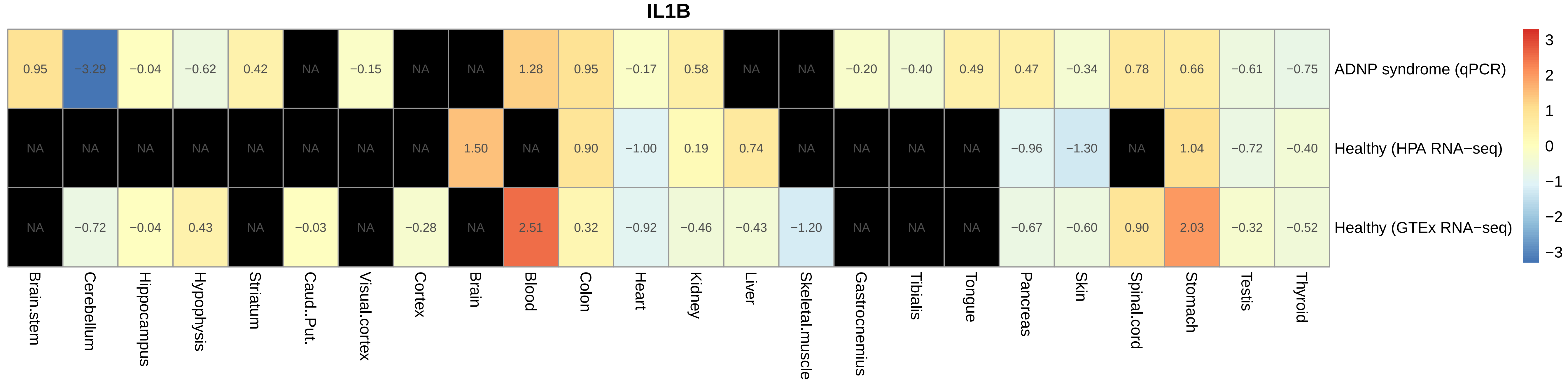
IL1B
0.95 −3.29 −0.04 −0.62 0.42 NA −0.15 NA NA 1.28 0.95 −0.17 0.58 NA NA −0.20 −0.40 0.49 0.47 −0.34 0.78 0.66 −0.61 −0.75
ADNP syndrome (qPCR)
NA NA NA NA NA NA NA NA 1.50 NA 0.90 −1.00 0.19 0.74 NA NA NA NA −0.96 −1.30 NA 1.04 −0.72 −0.40
Healthy (HPA RNA−seq)
NA −0.72 −0.04 0.43 NA −0.03 NA −0.28 NA 2.51 0.32 −0.92 −0.46 −0.43 −1.20 NA NA NA −0.67 −0.60 0.90 2.03 −0.32 −0.52
Healthy (GTEx RNA−seq)
3
2
1
0
−1
−2
−3
Brain.stem
Cerebellum
Hippocampus
Hypophysis
Striatum
Caud..Put.
Visual.cortex
Cortex
Brain
Blood
Colon
Heart
Kidney
Liver
Skeletal.muscle
Gastrocnemius
Tibialis
Tongue
Pancreas
Skin
Spinal.cord
Stomach
Testis
Thyroid

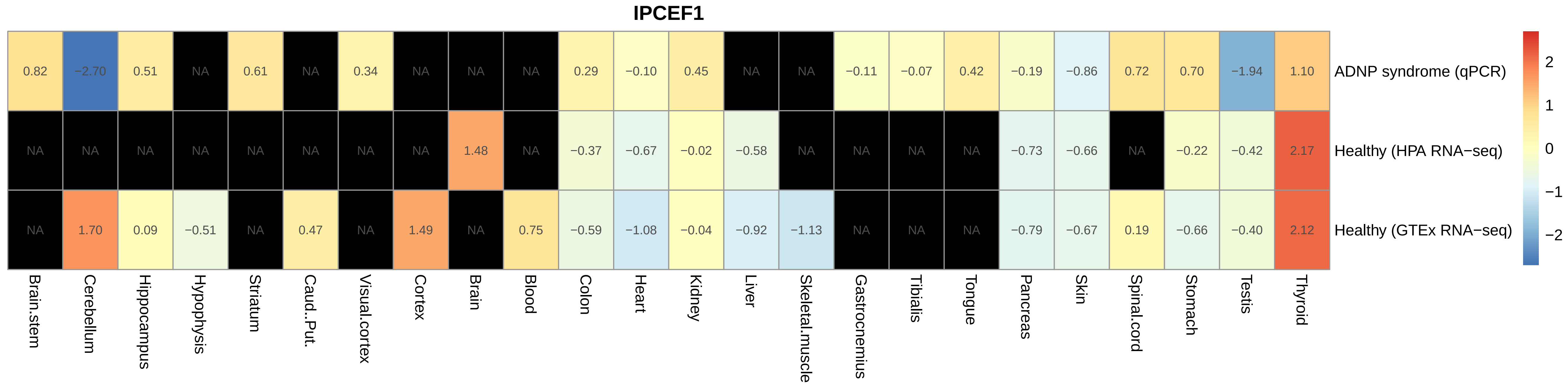
IPCEF1
0.82 −2.70 0.51 NA 0.61 NA 0.34 NA NA NA 0.29 −0.10 0.45 NA NA −0.11 −0.07 0.42 −0.19 −0.86 0.72 0.70 −1.94 1.10
ADNP syndrome (qPCR)
NA NA NA NA NA NA NA NA 1.48 NA −0.37 −0.67 −0.02 −0.58 NA NA NA NA −0.73 −0.66 NA −0.22 −0.42 2.17
Healthy (HPA RNA−seq)
NA 1.70 0.09 −0.51 NA 0.47 NA 1.49 NA 0.75 −0.59 −1.08 −0.04 −0.92 −1.13 NA NA NA −0.79 −0.67 0.19 −0.66 −0.40 2.12
Healthy (GTEx RNA−seq)
2
1
0
−1
−2
Brain.stem
Cerebellum
Hippocampus
Hypophysis
Striatum
Caud..Put.
Visual.cortex
Cortex
Brain
Blood
Colon
Heart
Kidney
Liver
Skeletal.muscle
Gastrocnemius
Tibialis
Tongue
Pancreas
Skin
Spinal.cord
Stomach
Testis
Thyroid

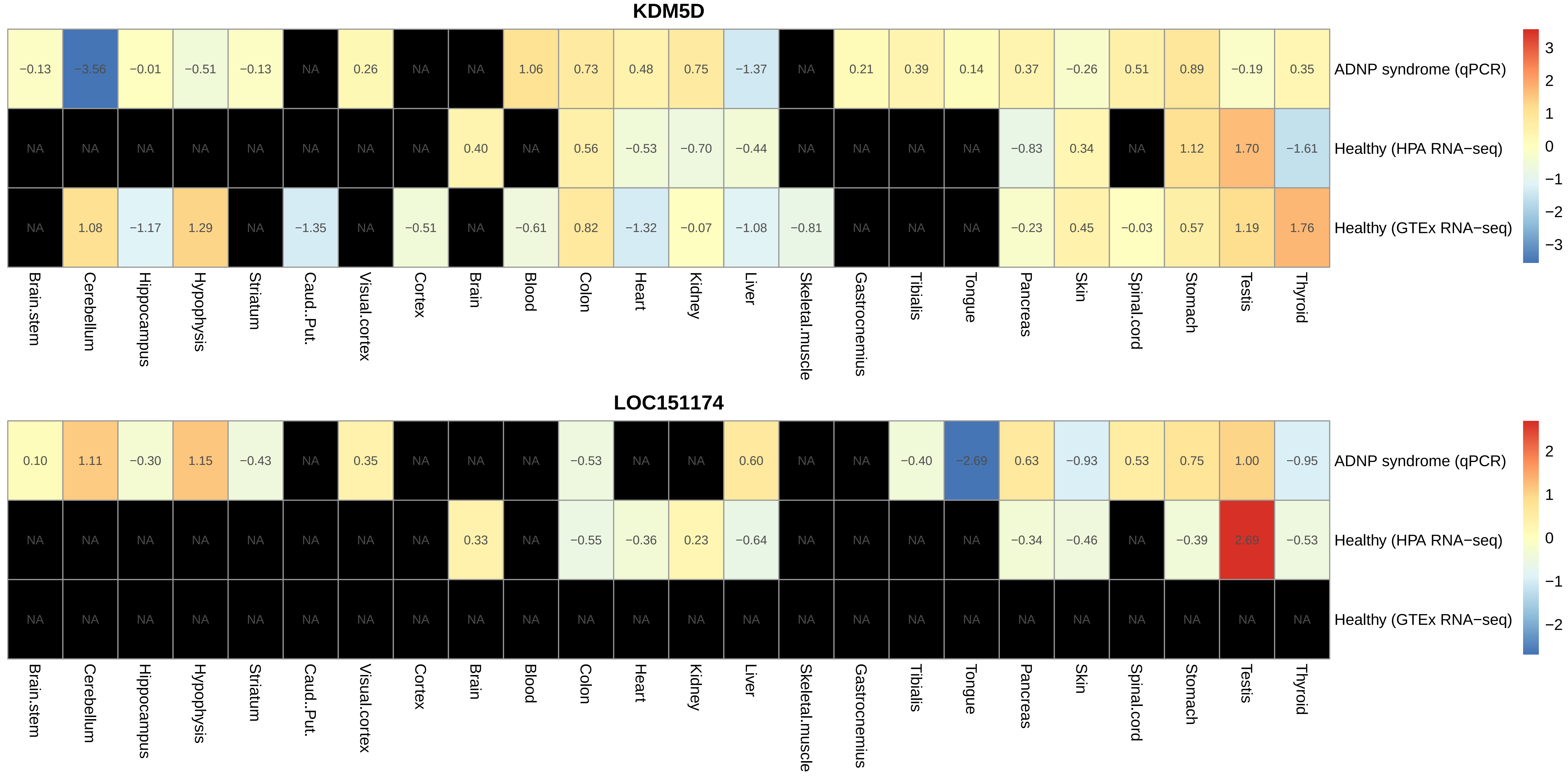
KDM5D
ADNP syndrome (qPCR)
−0.13 −3.56 −0.01 −0.51 −0.13 NA 0.26 NA NA 1.06 0.73 0.48 0.75 −1.37 NA 0.21 0.39 0.14 0.37 −0.26 0.51 0.89 −0.19 0.35
Healthy (HPA RNA−seq)
NA NA NA NA NA NA NA NA 0.40 NA 0.56 −0.53 −0.70 −0.44 NA NA NA NA −0.83 0.34 NA 1.12 1.70 −1.61
Healthy (GTEx RNA−seq)
NA 1.08 −1.17 1.29 NA −1.35 NA −0.51 NA −0.61 0.82 −1.32 −0.07 −1.08 −0.81 NA NA NA −0.23 0.45 −0.03 0.57 1.19 1.76
Brain.stem Cerebellum Hippocampus Hypophysis Striatum Caud..Put. Visual.cortex Cortex Brain Blood Colon Heart Kidney Liver Skeletal.muscle Gastrocnemius Tibialis Tongue Pancreas Skin Spinal.cord Stomach Testis Thyroid
3 2 1 0 −1 −2 −3
LOC151174
ADNP syndrome (qPCR)
0.10 1.11 −0.30 1.15 −0.43 NA 0.35 NA NA NA −0.53 NA NA 0.60 NA NA −0.40 −2.69 0.63 −0.93 0.53 0.75 1.00 −0.95
Healthy (HPA RNA−seq)
NA NA NA NA NA NA NA NA 0.33 NA −0.55 −0.36 0.23 −0.64 NA NA NA NA −0.34 −0.46 NA −0.39 2.69 −0.53
Healthy (GTEx RNA−seq)
NA NA NA NA NA NA NA NA NA NA NA NA NA NA NA NA NA NA NA NA NA NA NA NA
Brain.stem Cerebellum Hippocampus Hypophysis Striatum Caud..Put. Visual.cortex Cortex Brain Blood Colon Heart Kidney Liver Skeletal.muscle Gastrocnemius Tibialis Tongue Pancreas Skin Spinal.cord Stomach Testis Thyroid
2 1 0 −1 −2

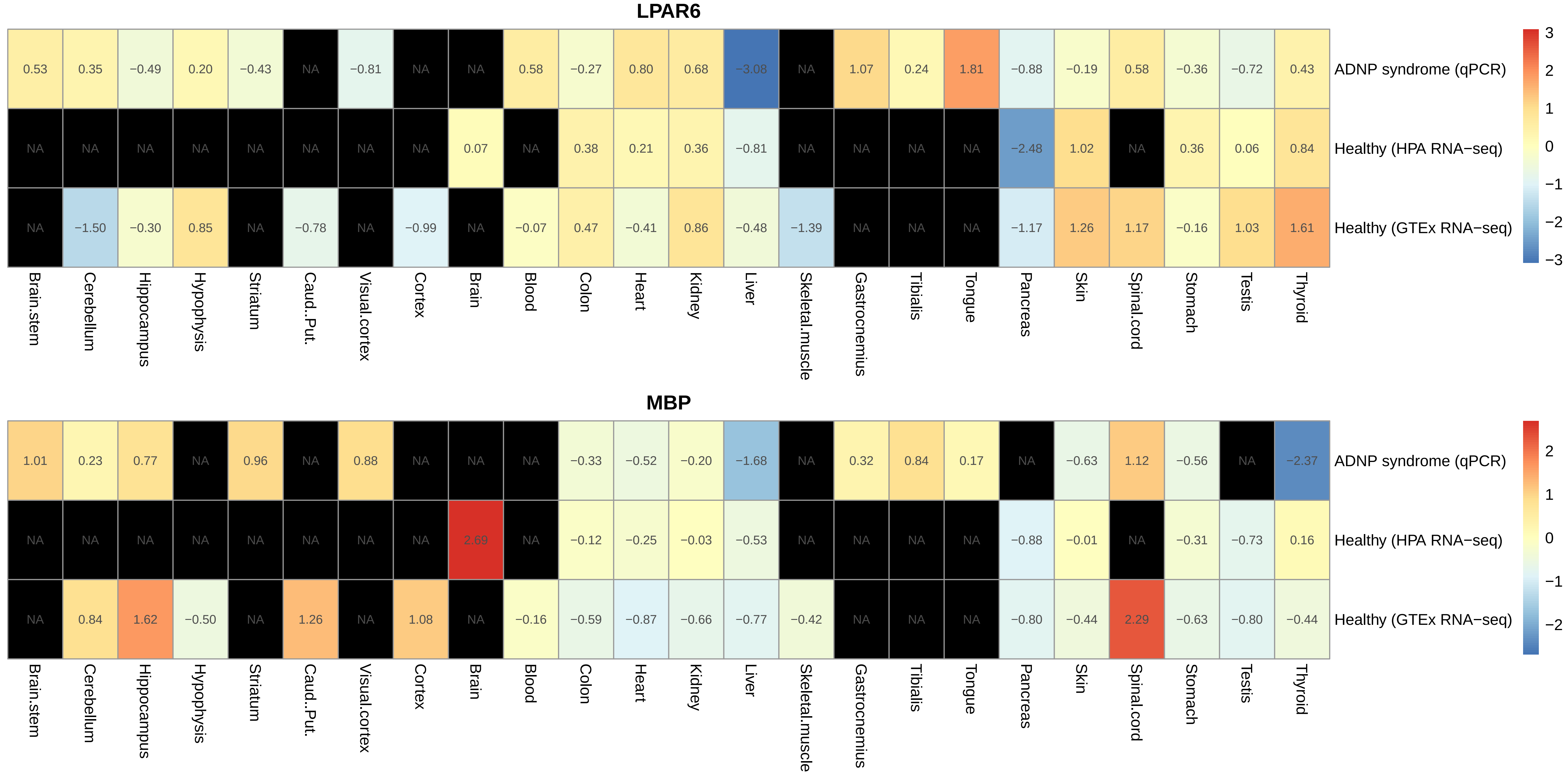
LPAR6
ADNP syndrome (qPCR)
Healthy (HPA RNA−seq)
Healthy (GTEx RNA−seq)
Brain.stem
Cerebellum
Hippocampus
Hypophysis
Striatum
Caud..Put.
Visual.cortex
Cortex
Brain
Blood
Colon
Heart
Kidney
Liver
Skeletal.muscle
Gastrocnemius
Tibialis
Tongue
Pancreas
Skin
Spinal.cord
Stomach
Testis
Thyroid
0.53 0.35 −0.49 0.20 −0.43 NA −0.81 NA NA 0.58 −0.27 0.80 0.68 −3.08 NA 1.07 0.24 1.81 −0.88 −0.19 0.58 −0.36 −0.72 0.43
NA NA NA NA NA NA NA NA 0.07 NA 0.38 0.21 0.36 −0.81 NA NA NA NA −2.48 1.02 NA 0.36 0.06 0.84
NA −1.50 −0.30 0.85 NA −0.78 NA −0.99 NA −0.07 0.47 −0.41 0.86 −0.48 −1.39 NA NA NA −1.17 1.26 1.17 −0.16 1.03 1.61
3
2
1
0
−1
−2
−3
MBP
ADNP syndrome (qPCR)
Healthy (HPA RNA−seq)
Healthy (GTEx RNA−seq)
Brain.stem
Cerebellum
Hippocampus
Hypophysis
Striatum
Caud..Put.
Visual.cortex
Cortex
Brain
Blood
Colon
Heart
Kidney
Liver
Skeletal.muscle
Gastrocnemius
Tibialis
Tongue
Pancreas
Skin
Spinal.cord
Stomach
Testis
Thyroid
1.01 0.23 0.77 NA 0.96 NA 0.88 NA NA NA −0.33 −0.52 −0.20 −1.68 NA 0.32 0.84 0.17 NA −0.63 1.12 −0.56 NA −2.37
NA NA NA NA NA NA NA NA 2.69 NA −0.12 −0.25 −0.03 −0.53 NA NA NA NA −0.88 −0.01 NA −0.31 −0.73 0.16
NA 0.84 1.62 −0.50 NA 1.26 NA 1.08 NA −0.16 −0.59 −0.87 −0.66 −0.77 −0.42 NA NA NA −0.80 −0.44 2.29 −0.63 −0.80 −0.44
2
1
0
−1
−2

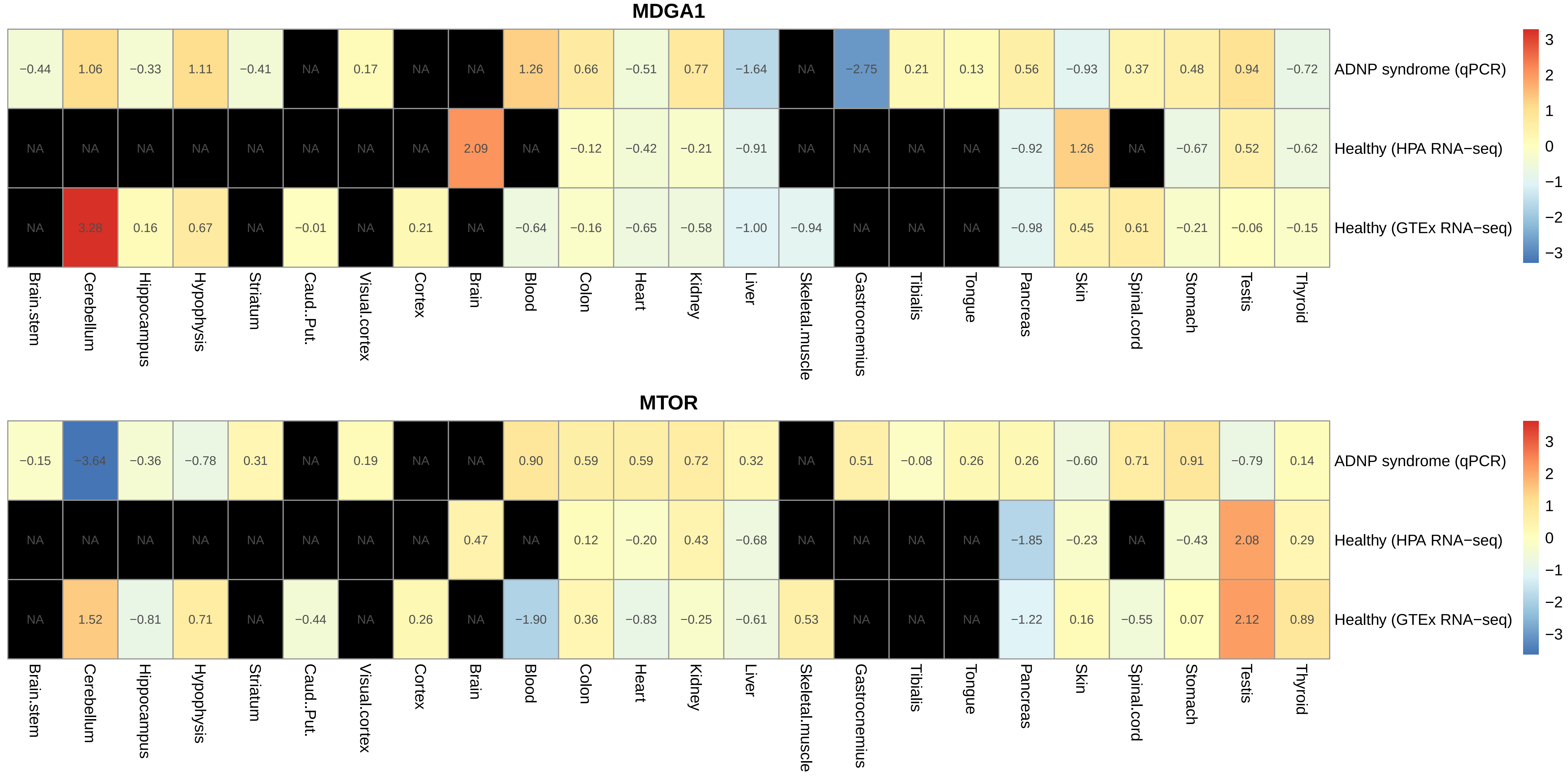

MDGA1
−0.44 1.06 −0.33 1.11 −0.41 NA 0.17 NA NA 1.26 0.66 −0.51 0.77 −1.64 NA −2.75 0.21 0.13 0.56 −0.93 0.37 0.48 0.94 −0.72
ADNP syndrome (qPCR)
NA NA NA NA NA NA NA NA 2.09 NA −0.12 −0.42 −0.21 −0.91 NA NA NA NA −0.92 1.26 NA −0.67 0.52 −0.62
Healthy (HPA RNA−seq)
NA 3.28 0.16 0.67 NA −0.01 NA 0.21 NA −0.64 −0.16 −0.65 −0.58 −1.00 −0.94 NA NA NA −0.98 0.45 0.61 −0.21 −0.06 −0.15
Healthy (GTEx RNA−seq)
Brain.stem
Cerebellum
Hippocampus
Hypophysis
Striatum
Caud..Put.
Visual.cortex
Cortex
Brain
Blood
Colon
Heart
Kidney
Liver
Skeletal.muscle
Gastrocnemius
Tibialis
Tongue
Pancreas
Skin
Spinal.cord
Stomach
Testis
Thyroid
3
2
1
0
−1
−2
−3
MTOR
−0.15 −3.64 −0.36 −0.78 0.31 NA 0.19 NA NA 0.90 0.59 0.59 0.72 0.32 NA 0.51 −0.08 0.26 0.26 −0.60 0.71 0.91 −0.79 0.14
ADNP syndrome (qPCR)
NA NA NA NA NA NA NA NA 0.47 NA 0.12 −0.20 0.43 −0.68 NA NA NA NA −1.85 −0.23 NA −0.43 2.08 0.29
Healthy (HPA RNA−seq)
NA 1.52 −0.81 0.71 NA −0.44 NA 0.26 NA −1.90 0.36 −0.83 −0.25 −0.61 0.53 NA NA NA −1.22 0.16 −0.55 0.07 2.12 0.89
Healthy (GTEx RNA−seq)
Brain.stem
Cerebellum
Hippocampus
Hypophysis
Striatum
Caud..Put.
Visual.cortex
Cortex
Brain
Blood
Colon
Heart
Kidney
Liver
Skeletal.muscle
Gastrocnemius
Tibialis
Tongue
Pancreas
Skin
Spinal.cord
Stomach
Testis
Thyroid
3
2
1
0
−1
−2
−3

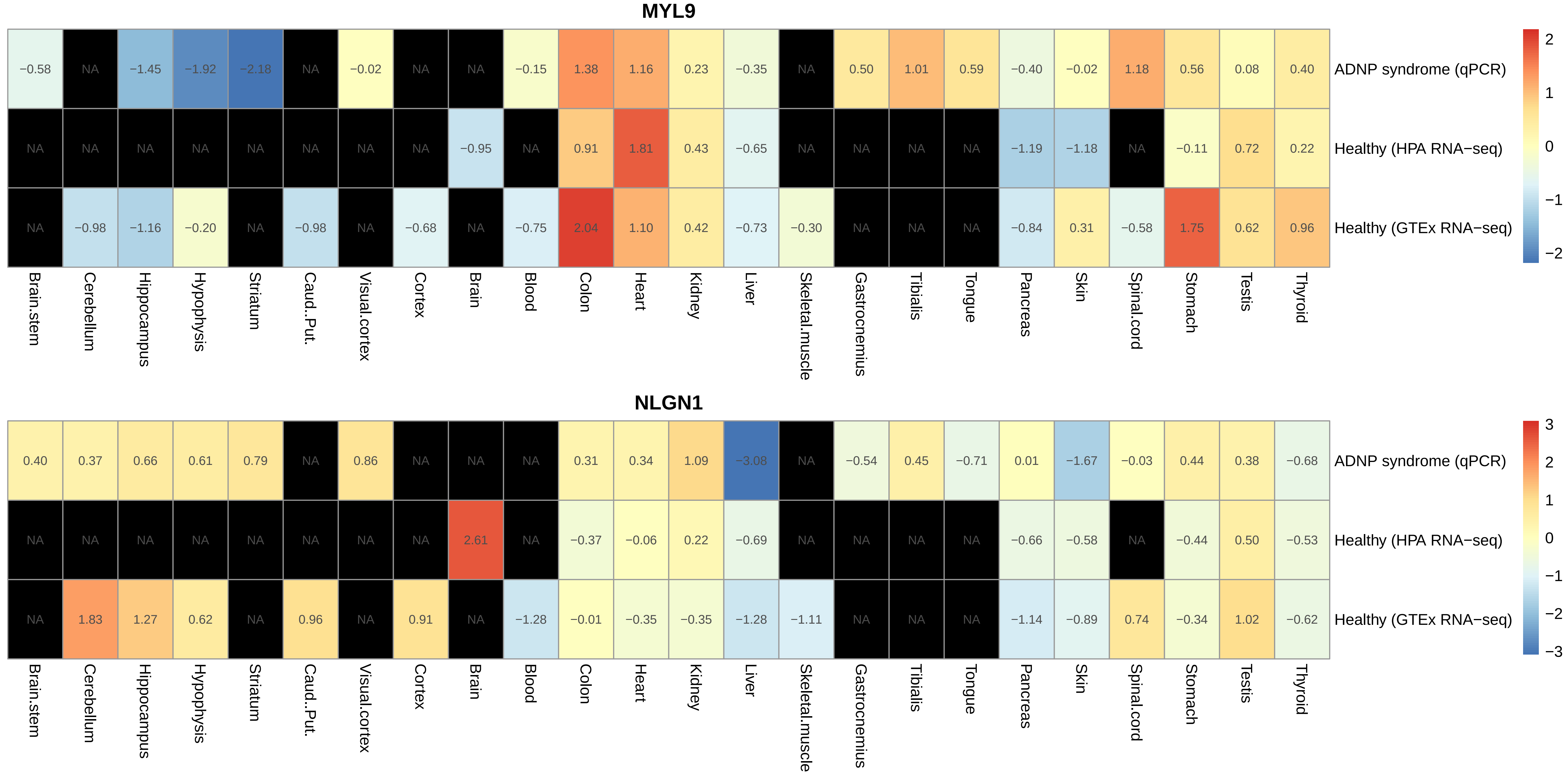
MYL9
−0.58 NA −1.45 −1.92 −2.18 NA −0.02 NA NA −0.15 1.38 1.16 0.23 −0.35 NA 0.50 1.01 0.59 −0.40 −0.02 1.18 0.56 0.08 0.40
ADNP syndrome (qPCR)
NA NA NA NA NA NA NA NA −0.95 NA 0.91 1.81 0.43 −0.65 NA NA NA NA −1.19 −1.18 NA −0.11 0.72 0.22
Healthy (HPA RNA−seq)
NA −0.98 −1.16 −0.20 NA −0.98 NA −0.68 NA −0.75 2.04 1.10 0.42 −0.73 −0.30 NA NA NA −0.84 0.31 −0.58 1.75 0.62 0.96
Healthy (GTEx RNA−seq)
2
1
0
−1
−2
Brain.stem
Cerebellum
Hippocampus
Hypophysis
Striatum
Caud..Put.
Visual.cortex
Cortex
Brain
Blood
Colon
Heart
Kidney
Liver
Skeletal.muscle
Gastrocnemius
Tibialis
Tongue
Pancreas
Skin
Spinal.cord
Stomach
Testis
Thyroid
NLGN1
0.40 0.37 0.66 0.61 0.79 NA 0.86 NA NA NA 0.31 0.34 1.09 −3.08 NA −0.54 0.45 −0.71 0.01 −1.67 −0.03 0.44 0.38 −0.68
ADNP syndrome (qPCR)
NA NA NA NA NA NA NA NA 2.61 NA −0.37 −0.06 0.22 −0.69 NA NA NA NA −0.66 −0.58 NA −0.44 0.50 −0.53
Healthy (HPA RNA−seq)
NA 1.83 1.27 0.62 NA 0.96 NA 0.91 NA −1.28 −0.01 −0.35 −0.35 −1.28 −1.11 NA NA NA −1.14 −0.89 0.74 −0.34 1.02 −0.62
Healthy (GTEx RNA−seq)
3
2
1
0
−1
−2
−3
Brain.stem
Cerebellum
Hippocampus
Hypophysis
Striatum
Caud..Put.
Visual.cortex
Cortex
Brain
Blood
Colon
Heart
Kidney
Liver
Skeletal.muscle
Gastrocnemius
Tibialis
Tongue
Pancreas
Skin
Spinal.cord
Stomach
Testis
Thyroid

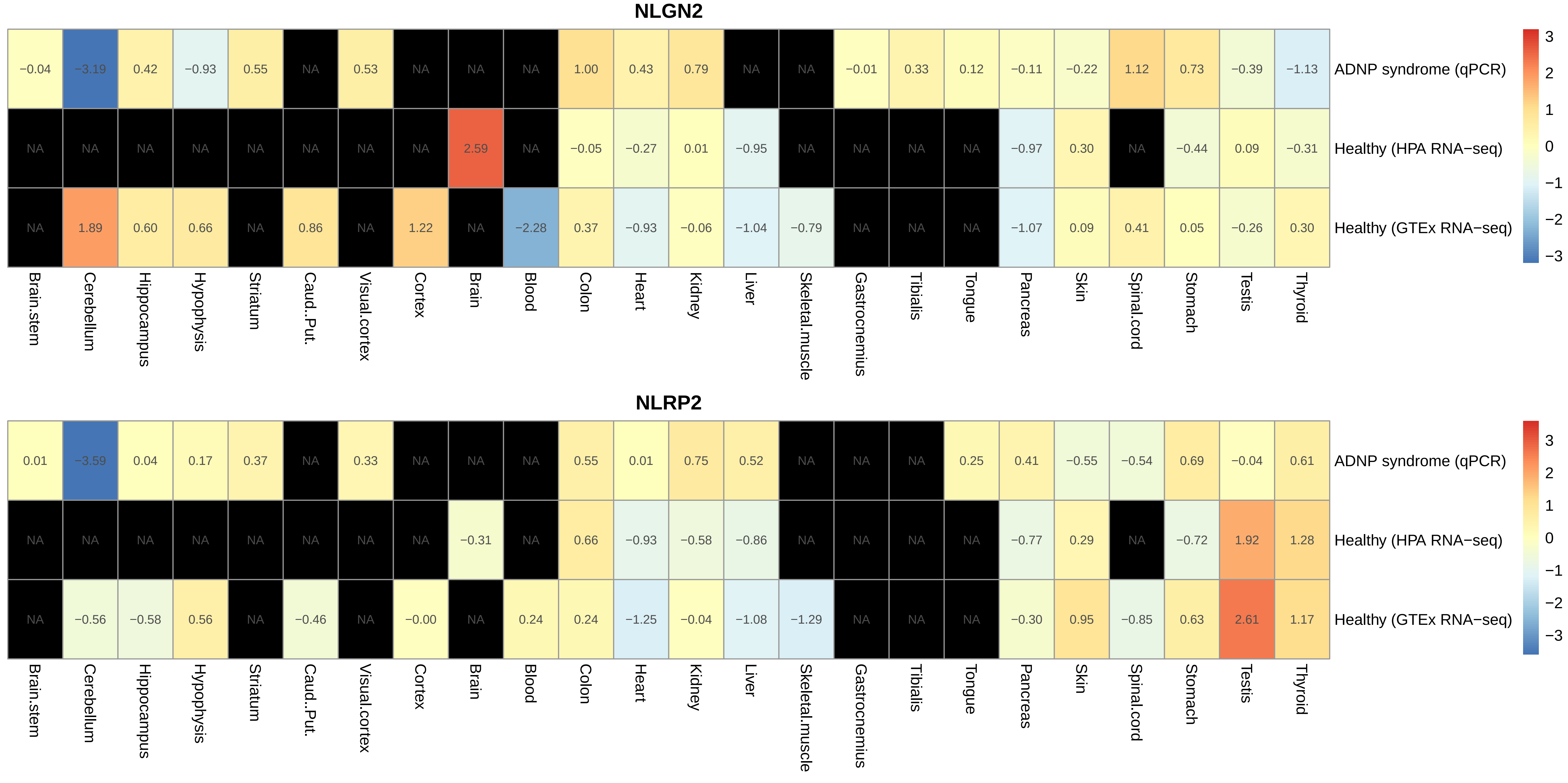
NLGN2
NLRP2
ADNP syndrome (qPCR)
Healthy (HPA RNA−seq)
Healthy (GTEx RNA−seq)
Brain.stem
Cerebellum
Hippocampus
Hypophysis
Striatum
Caud..Put.
Visual.cortex
Cortex
Brain
Blood
Colon
Heart
Kidney
Liver
Skeletal.muscle
Gastrocnemius
Tibialis
Tongue
Pancreas
Skin
Spinal.cord
Stomach
Testis
Thyroid

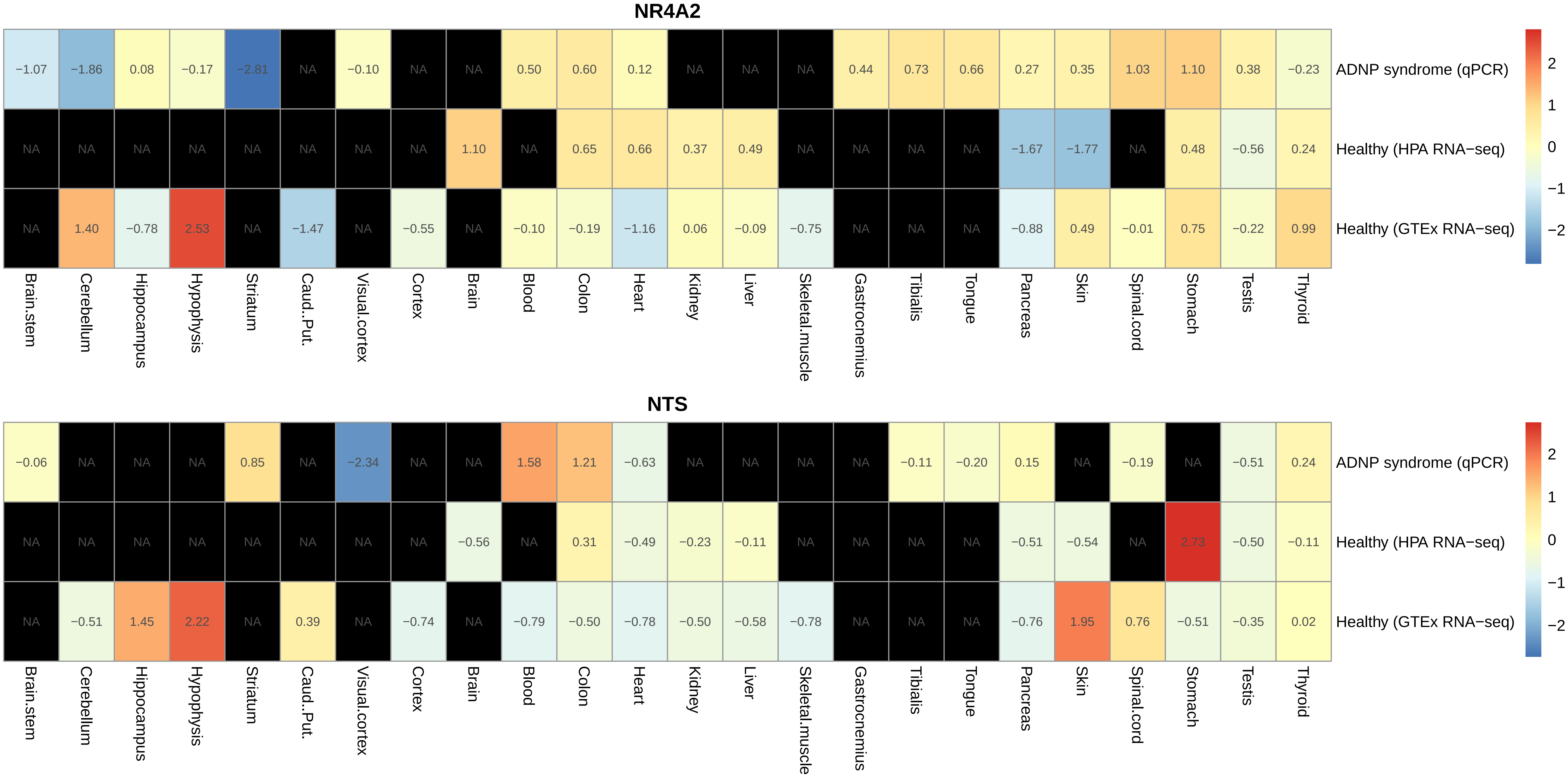
NR4A2
ADNP syndrome (qPCR)
−1.07 −1.86 0.08 −0.17 −2.81 NA −0.10 NA NA 0.50 0.60 0.12 NA NA NA 0.44 0.73 0.66 0.27 0.35 1.03 1.10 0.38 −0.23
Healthy (HPA RNA−seq)
NA NA NA NA NA NA NA NA 1.10 NA 0.65 0.66 0.37 0.49 NA NA NA NA −1.67 −1.77 NA 0.48 −0.56 0.24
Healthy (GTEx RNA−seq)
NA 1.40 −0.78 2.53 NA −1.47 NA −0.55 NA −0.10 −0.19 −1.16 0.06 −0.09 −0.75 NA NA NA −0.88 0.49 −0.01 0.75 −0.22 0.99
Brain.stem
Cerebellum
Hippocampus
Hypophysis
Striatum
Caud..Put.
Visual.cortex
Cortex
Brain
Blood
Colon
Heart
Kidney
Liver
Skeletal.muscle
Gastrocnemius
Tibialis
Tongue
Pancreas
Skin
Spinal.cord
Stomach
Testis
Thyroid
2
1
0
−1
−2
NTS
ADNP syndrome (qPCR)
−0.06 NA NA NA 0.85 NA −2.34 NA NA 1.58 1.21 −0.63 NA NA NA NA −0.11 −0.20 0.15 NA −0.19 NA −0.51 0.24
Healthy (HPA RNA−seq)
NA NA NA NA NA NA NA NA −0.56 NA 0.31 −0.49 −0.23 −0.11 NA NA NA NA −0.51 −0.54 NA 2.73 −0.50 −0.11
Healthy (GTEx RNA−seq)
NA −0.51 1.45 2.22 NA 0.39 NA −0.74 NA −0.79 −0.50 −0.78 −0.50 −0.58 −0.78 NA NA NA −0.76 1.95 0.76 −0.51 −0.35 0.02
Brain.stem
Cerebellum
Hippocampus
Hypophysis
Striatum
Caud..Put.
Visual.cortex
Cortex
Brain
Blood
Colon
Heart
Kidney
Liver
Skeletal.muscle
Gastrocnemius
Tibialis
Tongue
Pancreas
Skin
Spinal.cord
Stomach
Testis
Thyroid
2
1
0
−1
−2

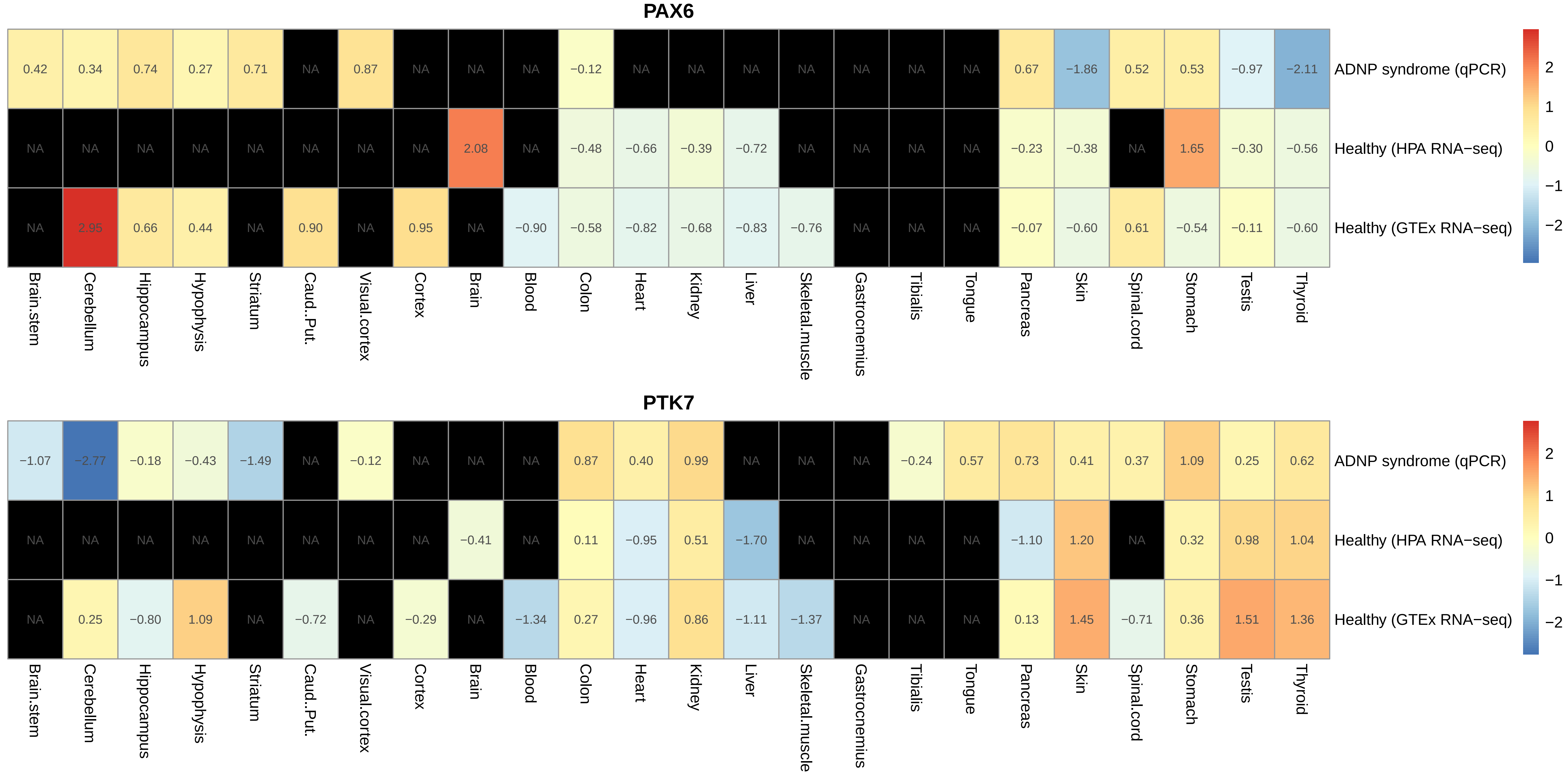
PAX6
0.42 0.34 0.74 0.27 0.71 NA 0.87 NA NA NA −0.12 NA NA NA NA NA NA NA 0.67 −1.86 0.52 0.53 −0.97 −2.11
ADNP syndrome (qPCR)
NA NA NA NA NA NA NA NA 2.08 NA −0.48 −0.66 −0.39 −0.72 NA NA NA NA −0.23 −0.38 NA 1.65 −0.30 −0.56
Healthy (HPA RNA−seq)
NA 2.95 0.66 0.44 NA 0.90 NA 0.95 NA −0.90 −0.58 −0.82 −0.68 −0.83 −0.76 NA NA NA −0.07 −0.60 0.61 −0.54 −0.11 −0.60
Healthy (GTEx RNA−seq)
Brain.stem
Cerebellum
Hippocampus
Hypophysis
Striatum
Caud..Put.
Visual.cortex
Cortex
Brain
Blood
Colon
Heart
Kidney
Liver
Skeletal.muscle
Gastrocnemius
Tibialis
Tongue
Pancreas
Skin
Spinal.cord
Stomach
Testis
Thyroid
2
1
0
−1
−2
PTK7
−1.07 −2.77 −0.18 −0.43 −1.49 NA −0.12 NA NA NA 0.87 0.40 0.99 NA NA NA −0.24 0.57 0.73 0.41 0.37 1.09 0.25 0.62
ADNP syndrome (qPCR)
NA NA NA NA NA NA NA NA −0.41 NA 0.11 −0.95 0.51 −1.70 NA NA NA NA −1.10 1.20 NA 0.32 0.98 1.04
Healthy (HPA RNA−seq)
NA 0.25 −0.80 1.09 NA −0.72 NA −0.29 NA −1.34 0.27 −0.96 0.86 −1.11 −1.37 NA NA NA 0.13 1.45 −0.71 0.36 1.51 1.36
Healthy (GTEx RNA−seq)
Brain.stem
Cerebellum
Hippocampus
Hypophysis
Striatum
Caud..Put.
Visual.cortex
Cortex
Brain
Blood
Colon
Heart
Kidney
Liver
Skeletal.muscle
Gastrocnemius
Tibialis
Tongue
Pancreas
Skin
Spinal.cord
Stomach
Testis
Thyroid
2
1
0
−1
−2

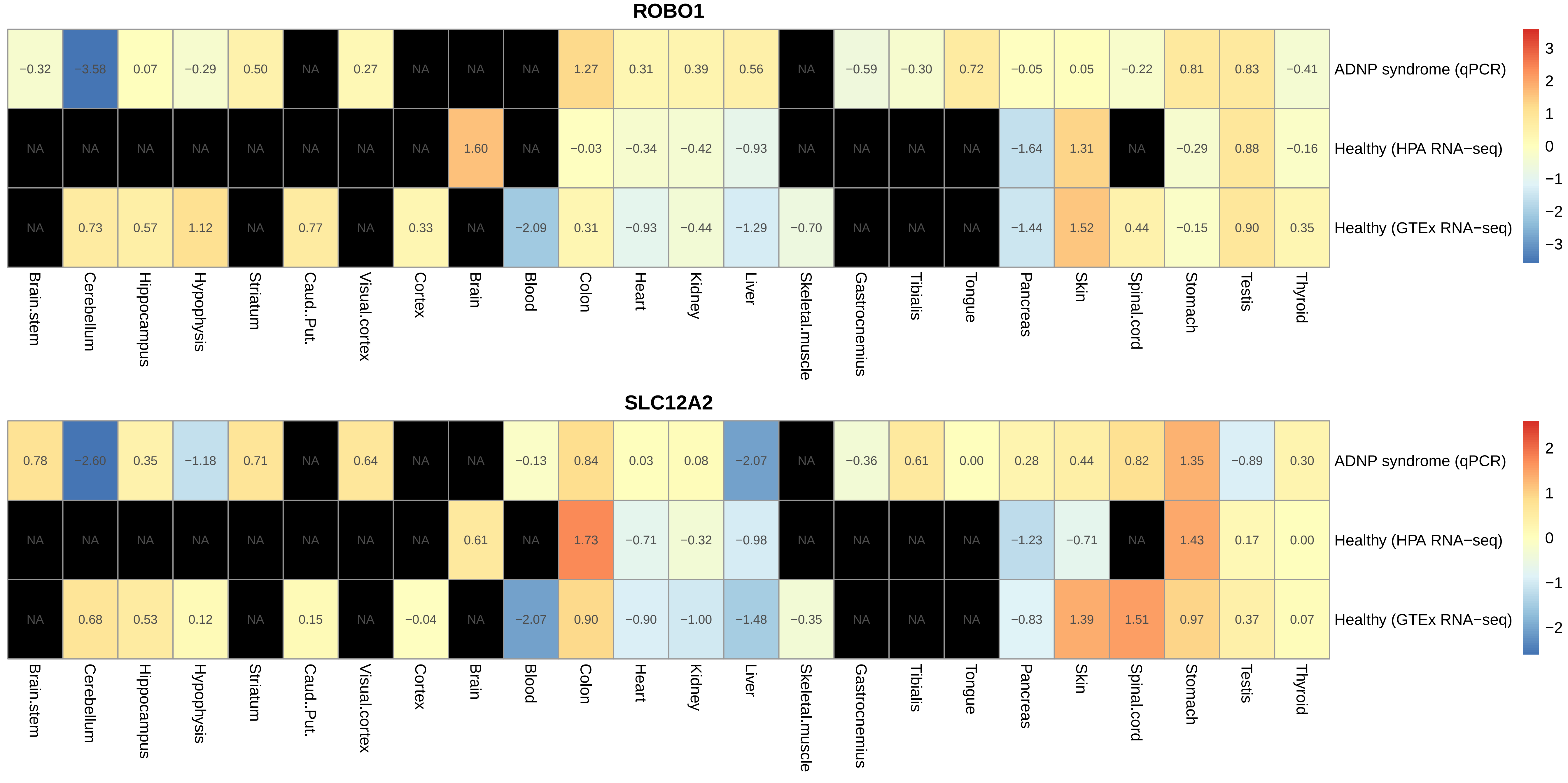
ROBO1
ADNP syndrome (qPCR)
Healthy (HPA RNA−seq)
Healthy (GTEx RNA−seq)
Brain.stem Cerebellum Hippocampus Hypophysis Striatum Caud..Put. Visual.cortex Cortex Brain Blood Colon Heart Kidney Liver Skeletal.muscle Gastrocnemius Tibialis Tongue Pancreas Skin Spinal.cord Stomach Testis Thyroid
−0.32 −3.58 0.07 −0.29 0.50 NA 0.27 NA NA NA 1.27 0.31 0.39 0.56 NA −0.59 −0.30 0.72 −0.05 0.05 −0.22 0.81 0.83 −0.41
NA NA NA NA NA NA NA NA 1.60 NA −0.03 −0.34 −0.42 −0.93 NA NA NA NA −1.64 1.31 NA −0.29 0.88 −0.16
NA 0.73 0.57 1.12 NA 0.77 NA 0.33 NA −2.09 0.31 −0.93 −0.44 −1.29 −0.70 NA NA NA −1.44 1.52 0.44 −0.15 0.90 0.35
3 2 1 0 −1 −2 −3
SLC12A2
ADNP syndrome (qPCR)
Healthy (HPA RNA−seq)
Healthy (GTEx RNA−seq)
Brain.stem Cerebellum Hippocampus Hypophysis Striatum Caud..Put. Visual.cortex Cortex Brain Blood Colon Heart Kidney Liver Skeletal.muscle Gastrocnemius Tibialis Tongue Pancreas Skin Spinal.cord Stomach Testis Thyroid
0.78 −2.60 0.35 −1.18 0.71 NA 0.64 NA NA −0.13 0.84 0.03 0.08 −2.07 NA −0.36 0.61 0.00 0.28 0.44 0.82 1.35 −0.89 0.30
NA NA NA NA NA NA NA NA 0.61 NA 1.73 −0.71 −0.32 −0.98 NA NA NA NA −1.23 −0.71 NA 1.43 0.17 0.00
NA 0.68 0.53 0.12 NA 0.15 NA −0.04 NA −2.07 0.90 −0.90 −1.00 −1.48 −0.35 NA NA NA −0.83 1.39 1.51 0.97 0.37 0.07
2 1 0 −1 −2

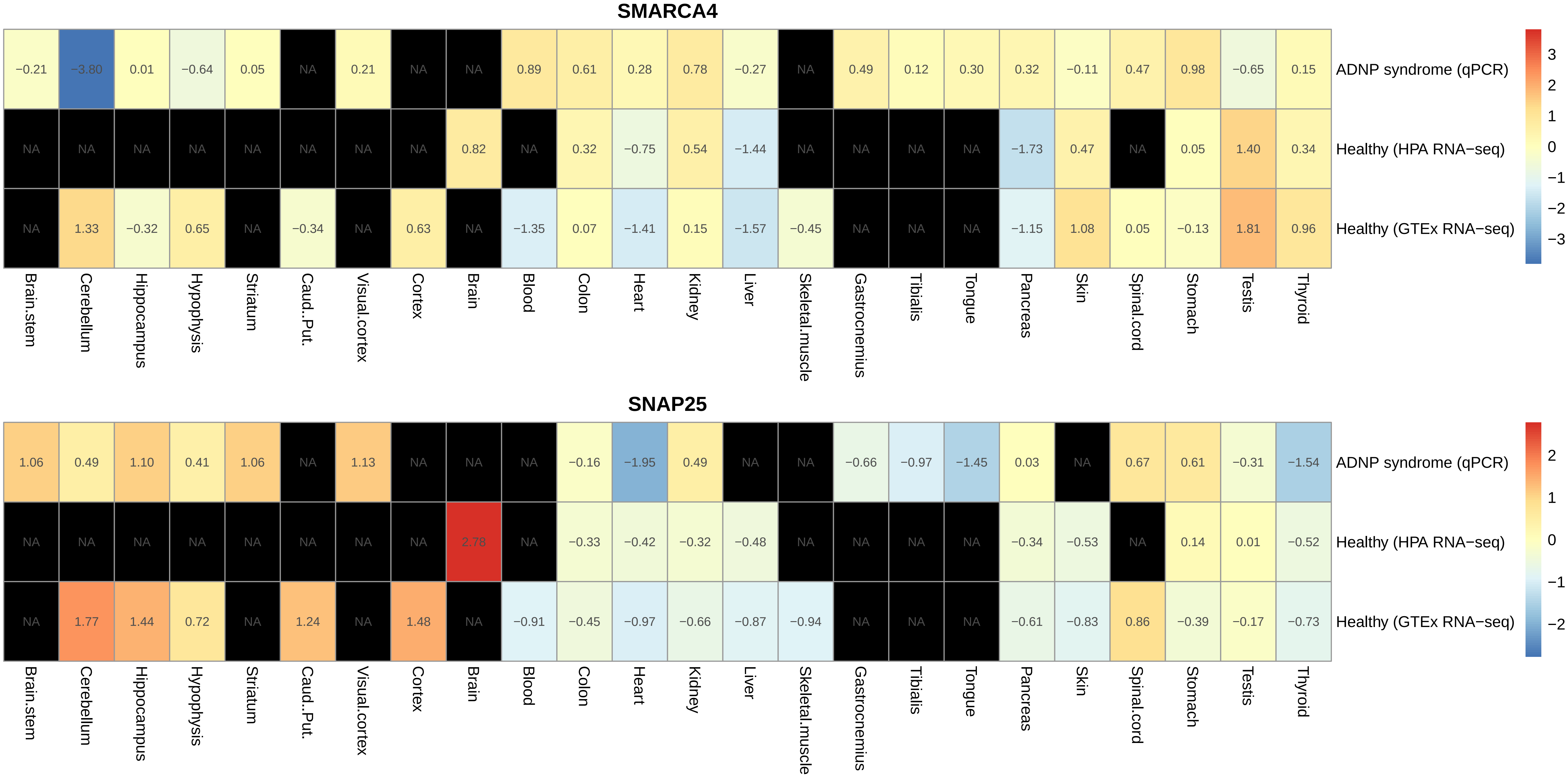
SMARCA4
ADNP syndrome (qPCR)
−0.21 −3.80 0.01 −0.64 0.05 NA 0.21 NA NA 0.89 0.61 0.28 0.78 −0.27 NA 0.49 0.12 0.30 0.32 −0.11 0.47 0.98 −0.65 0.15
Healthy (HPA RNA−seq)
NA NA NA NA NA NA NA NA 0.82 NA 0.32 −0.75 0.54 −1.44 NA NA NA NA −1.73 0.47 NA 0.05 1.40 0.34
Healthy (GTEx RNA−seq)
NA 1.33 −0.32 0.65 NA −0.34 NA 0.63 NA −1.35 0.07 −1.41 0.15 −1.57 −0.45 NA NA NA −1.15 1.08 0.05 −0.13 1.81 0.96
Brain.stem
Cerebellum
Hippocampus
Hypophysis
Striatum
Caud..Put.
Visual.cortex
Cortex
Brain
Blood
Colon
Heart
Kidney
Liver
Skeletal.muscle
Gastrocnemius
Tibialis
Tongue
Pancreas
Skin
Spinal.cord
Stomach
Testis
Thyroid
3
2
1
0
−1
−2
−3
SNAP25
ADNP syndrome (qPCR)
1.06 0.49 1.10 0.41 1.06 NA 1.13 NA NA NA −0.16 −1.95 0.49 NA NA −0.66 −0.97 −1.45 0.03 NA 0.67 0.61 −0.31 −1.54
Healthy (HPA RNA−seq)
NA NA NA NA NA NA NA NA 2.78 NA −0.33 −0.42 −0.32 −0.48 NA NA NA NA −0.34 −0.53 NA 0.14 0.01 −0.52
Healthy (GTEx RNA−seq)
NA 1.77 1.44 0.72 NA 1.24 NA 1.48 NA −0.91 −0.45 −0.97 −0.66 −0.87 −0.94 NA NA NA −0.61 −0.83 0.86 −0.39 −0.17 −0.73
Brain.stem
Cerebellum
Hippocampus
Hypophysis
Striatum
Caud..Put.
Visual.cortex
Cortex
Brain
Blood
Colon
Heart
Kidney
Liver
Skeletal.muscle
Gastrocnemius
Tibialis
Tongue
Pancreas
Skin
Spinal.cord
Stomach
Testis
Thyroid
2
1
0
−1
−2

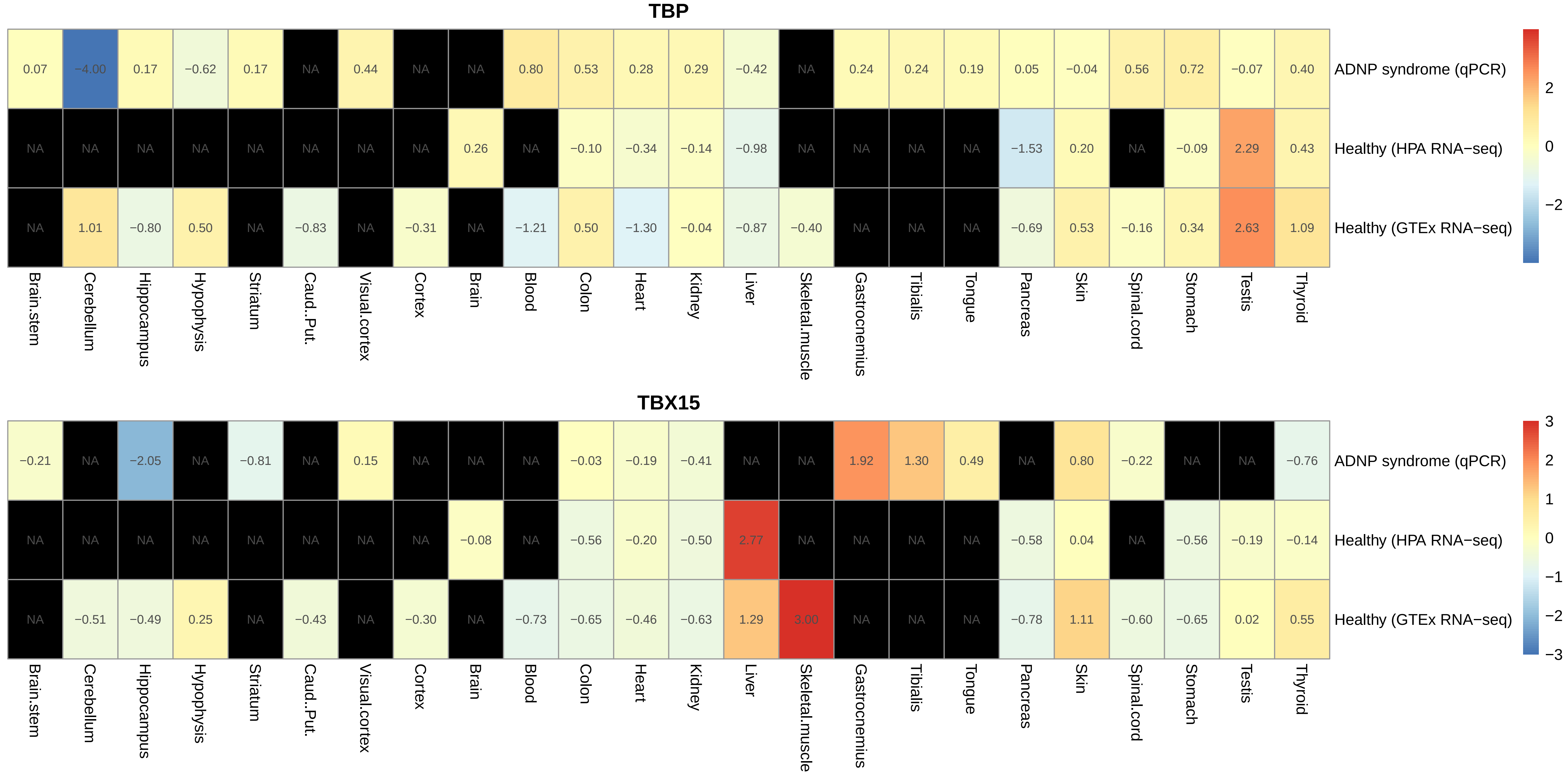
TBP
ADNP syndrome (qPCR)
0.07 −4.00 0.17 −0.62 0.17 NA 0.44 NA NA 0.80 0.53 0.28 0.29 −0.42 NA 0.24 0.24 0.19 0.05 −0.04 0.56 0.72 −0.07 0.40
Healthy (HPA RNA−seq)
NA NA NA NA NA NA NA NA 0.26 NA −0.10 −0.34 −0.14 −0.98 NA NA NA NA −1.53 0.20 NA −0.09 2.29 0.43
Healthy (GTEx RNA−seq)
NA 1.01 −0.80 0.50 NA −0.83 NA −0.31 NA −1.21 0.50 −1.30 −0.04 −0.87 −0.40 NA NA NA −0.69 0.53 −0.16 0.34 2.63 1.09
Brain.stem Cerebellum Hippocampus Hypophysis Striatum Caud..Put. Visual.cortex Cortex Brain Blood Colon Heart Kidney Liver Skeletal.muscle Gastrocnemius Tibialis Tongue Pancreas Skin Spinal.cord Stomach Testis Thyroid
2
0
−2
TBX15
ADNP syndrome (qPCR)
−0.21 NA −2.05 NA −0.81 NA 0.15 NA NA NA −0.03 −0.19 −0.41 NA NA 1.92 1.30 0.49 NA 0.80 −0.22 NA NA −0.76
Healthy (HPA RNA−seq)
NA NA NA NA NA NA NA NA −0.08 NA −0.56 −0.20 −0.50 2.77 NA NA NA NA −0.58 0.04 NA −0.56 −0.19 −0.14
Healthy (GTEx RNA−seq)
NA −0.51 −0.49 0.25 NA −0.43 NA −0.30 NA −0.73 −0.65 −0.46 −0.63 1.29 3.00 NA NA NA −0.78 1.11 −0.60 −0.65 0.02 0.55
Brain.stem Cerebellum Hippocampus Hypophysis Striatum Caud..Put. Visual.cortex Cortex Brain Blood Colon Heart Kidney Liver Skeletal.muscle Gastrocnemius Tibialis Tongue Pancreas Skin Spinal.cord Stomach Testis Thyroid
3
2
1
0
−1
−2
−3

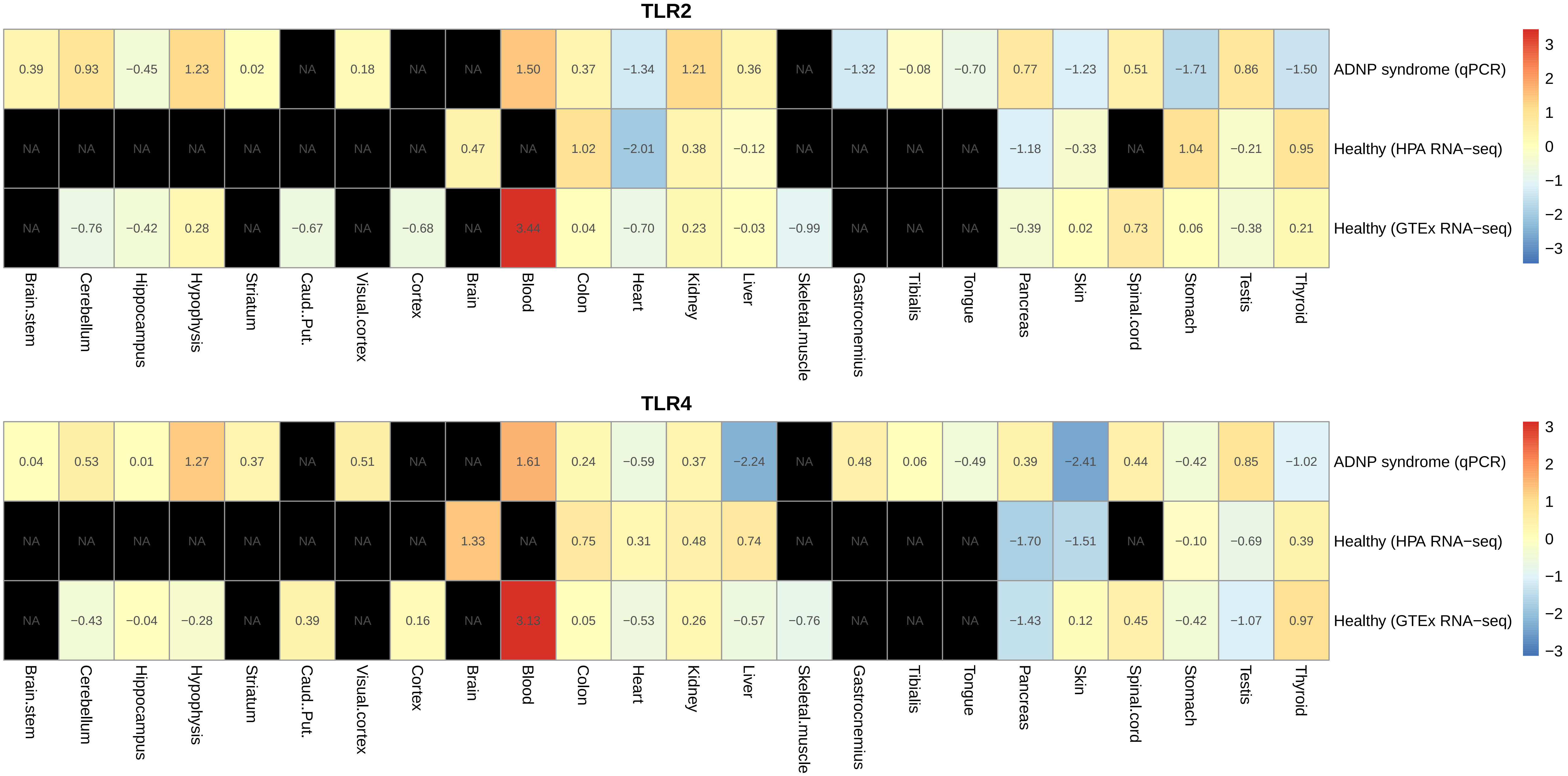
TLR2
ADNP syndrome (qPCR)
0.39 0.93 −0.45 1.23 0.02 NA 0.18 NA NA 1.50 0.37 −1.34 1.21 0.36 NA −1.32 −0.08 −0.70 0.77 −1.23 0.51 −1.71 0.86 −1.50
Healthy (HPA RNA−seq)
NA NA NA NA NA NA NA NA 0.47 NA 1.02 −2.01 0.38 −0.12 NA NA NA NA −1.18 −0.33 NA 1.04 −0.21 0.95
Healthy (GTEx RNA−seq)
NA −0.76 −0.42 0.28 NA −0.67 NA −0.68 NA 3.44 0.04 −0.70 0.23 −0.03 −0.99 NA NA NA −0.39 0.02 0.73 0.06 −0.38 0.21
Brain.stem
Cerebellum
Hippocampus
Hypophysis
Striatum
Caud..Put.
Visual.cortex
Cortex
Brain
Blood
Colon
Heart
Kidney
Liver
Skeletal.muscle
Gastrocnemius
Tibialis
Tongue
Pancreas
Skin
Spinal.cord
Stomach
Testis
Thyroid
3
2
1
0
−1
−2
−3
TLR4
ADNP syndrome (qPCR)
0.04 0.53 0.01 1.27 0.37 NA 0.51 NA NA 1.61 0.24 −0.59 0.37 −2.24 NA 0.48 0.06 −0.49 0.39 −2.41 0.44 −0.42 0.85 −1.02
Healthy (HPA RNA−seq)
NA NA NA NA NA NA NA NA 1.33 NA 0.75 0.31 0.48 0.74 NA NA NA NA −1.70 −1.51 NA −0.10 −0.69 0.39
Healthy (GTEx RNA−seq)
NA −0.43 −0.04 −0.28 NA 0.39 NA 0.16 NA 3.13 0.05 −0.53 0.26 −0.57 −0.76 NA NA NA −1.43 0.12 0.45 −0.42 −1.07 0.97
Brain.stem
Cerebellum
Hippocampus
Hypophysis
Striatum
Caud..Put.
Visual.cortex
Cortex
Brain
Blood
Colon
Heart
Kidney
Liver
Skeletal.muscle
Gastrocnemius
Tibialis
Tongue
Pancreas
Skin
Spinal.cord
Stomach
Testis
Thyroid
3
2
1
0
−1
−2
−3

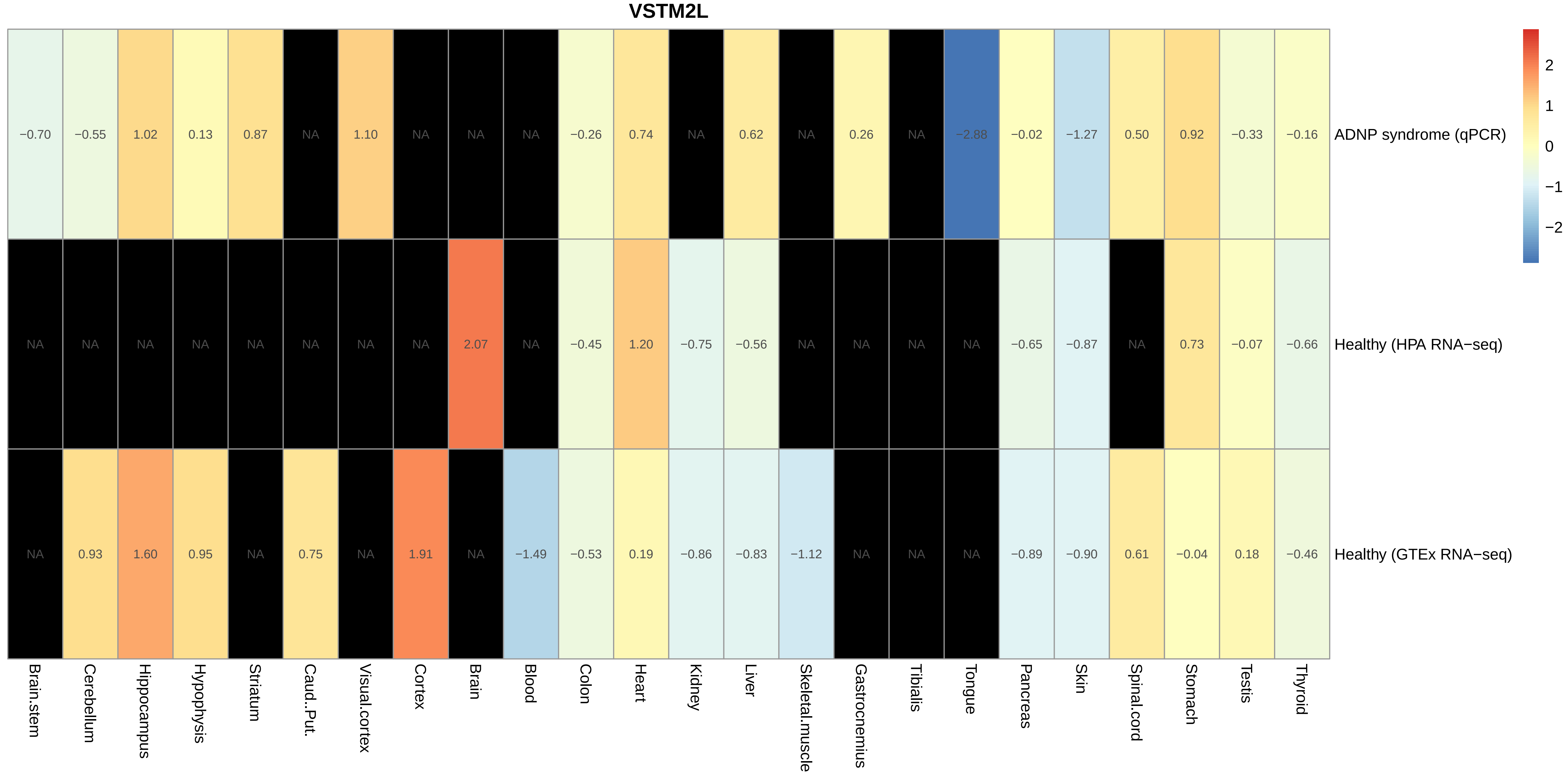
VSTM2L
ADNP syndrome (qPCR)
−0.70 −0.55 1.02 0.13 0.87 NA 1.10 NA NA NA −0.26 0.74 NA 0.62 NA 0.26 NA −2.88 −0.02 −1.27 0.50 0.92 −0.33 −0.16
Healthy (HPA RNA−seq)
NA NA NA NA NA NA NA NA 2.07 NA −0.45 1.20 −0.75 −0.56 NA NA NA NA −0.65 −0.87 NA 0.73 −0.07 −0.66
Healthy (GTEx RNA−seq)
NA 0.93 1.60 0.95 NA 0.75 NA 1.91 NA −1.49 −0.53 0.19 −0.86 −0.83 −1.12 NA NA NA −0.89 −0.90 0.61 −0.04 0.18 −0.46
Brain.stem
Cerebellum
Hippocampus
Hypophysis
Striatum
Caud..Put.
Visual.cortex
Cortex
Brain
Blood
Colon
Heart
Kidney
Liver
Skeletal.muscle
Gastrocnemius
Tibialis
Tongue
Pancreas
Skin
Spinal.cord
Stomach
Testis
Thyroid
2
1
0
−1
−2

Figure S3

Distributions of ADNP syndrome, HPA, and GTEx expression values

Each plot presents the density of the gene expression values (log2 transformed). Top (ADNP syndrome), middle (HPA dataset), and bottom (GTEx dataset).

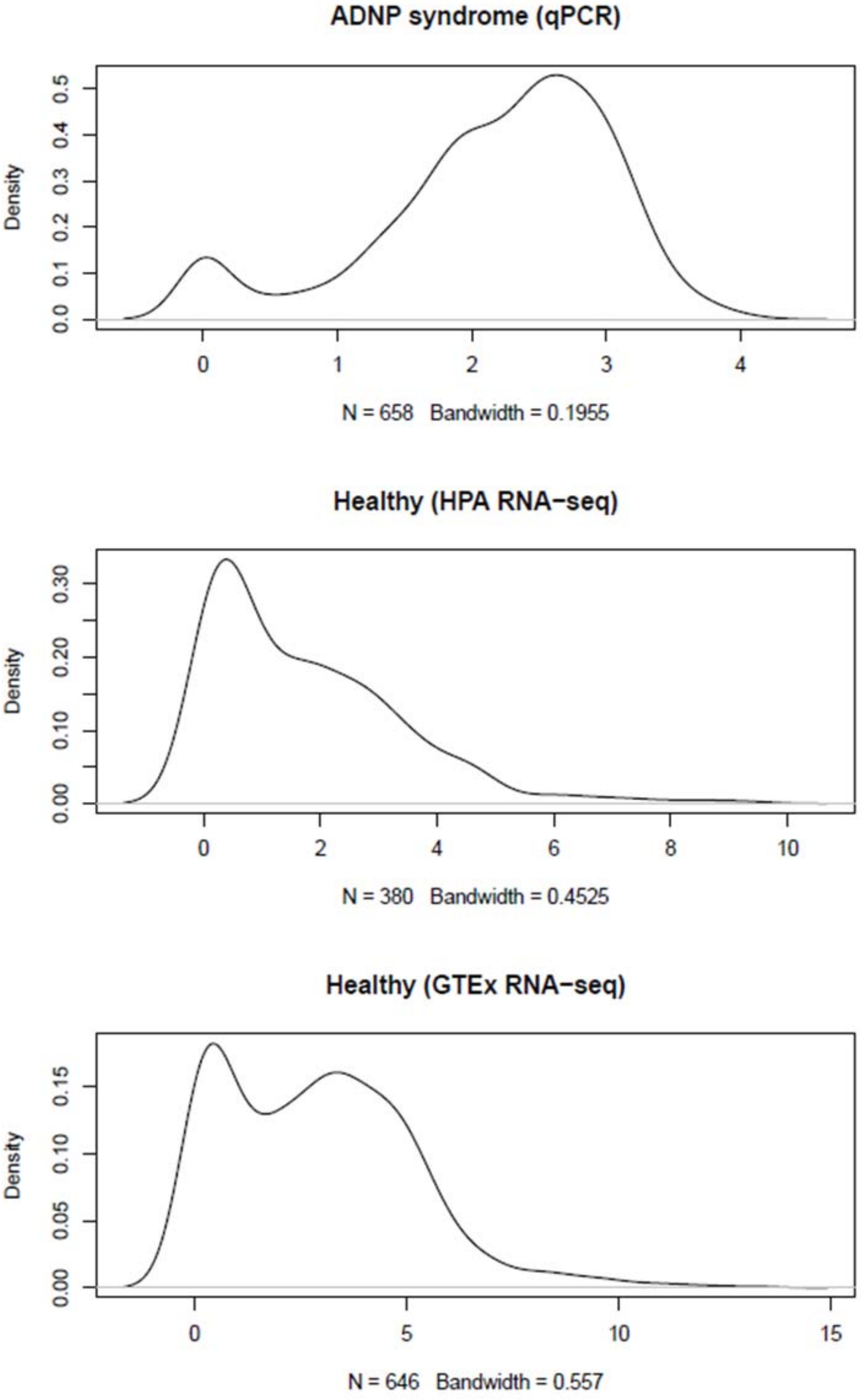

Figure S4

The ADNP syndrome olfactory bulb and hypothalamus exhibit intensive tauopathy, not detected in the corpus callosum and the trigeminal nerve

Gallyas staining (Tau pathology) is indicated by green arrows.

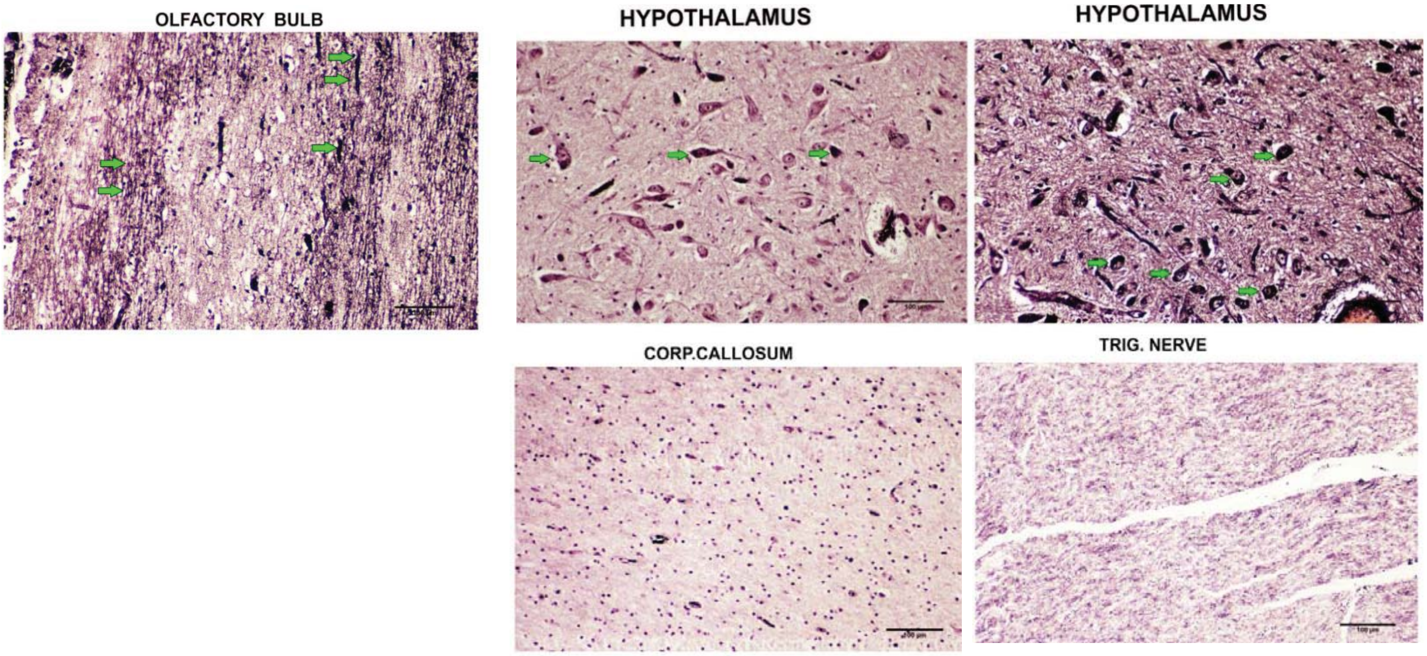

Figure S5A

Plasmid maps

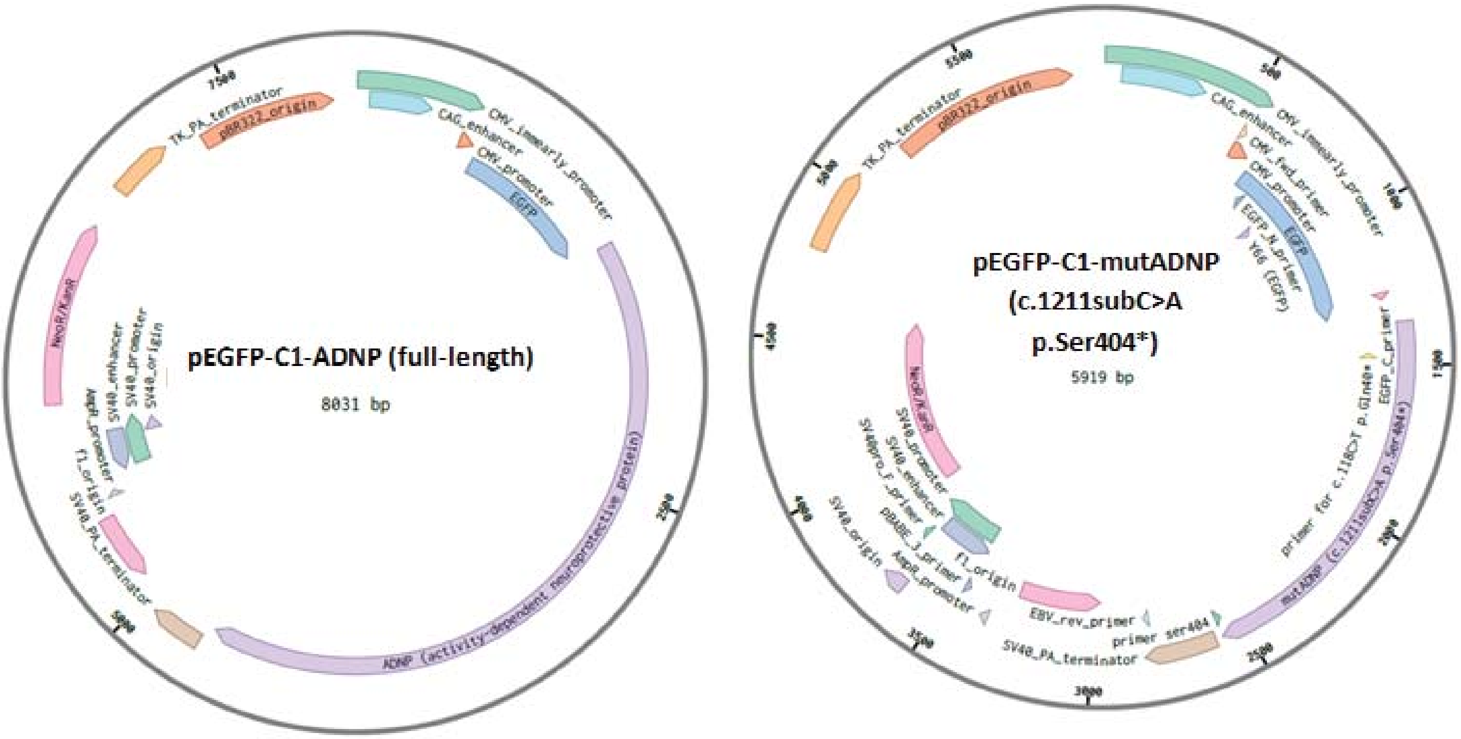

Protein expressing plasmids were based on pEGFP-C1 vector and express full-length ADNP or truncated form of ADNP. The plasmid maps were constructed with Benchling platform (www.benchling.com).

Figure S5B

Verification of plasmid expression

Verification of protein expression by fluorescent imaging. Differentiated N1E-115 cells were transfected with constructed plasmids (described in Fig. S2B). Pictures were taken by fluorescent microscope 48hrs after transfection (left). Verification of ADNP truncated protein expression by Western blotting. HEK293T cells were transfected with constructed plasmids (Fig. S2B), and whole-cell lysate proteins were extracted 48hrs after transfection. Blots were exposed to GFP-antibody. Expected molecular weight of GFP-conjugated proteins: GFP – 26.9kDa, GFP-full-length ADNP – 151.5kDa, GFP-mutADNP-p.Ser404* - 73.2kDa.

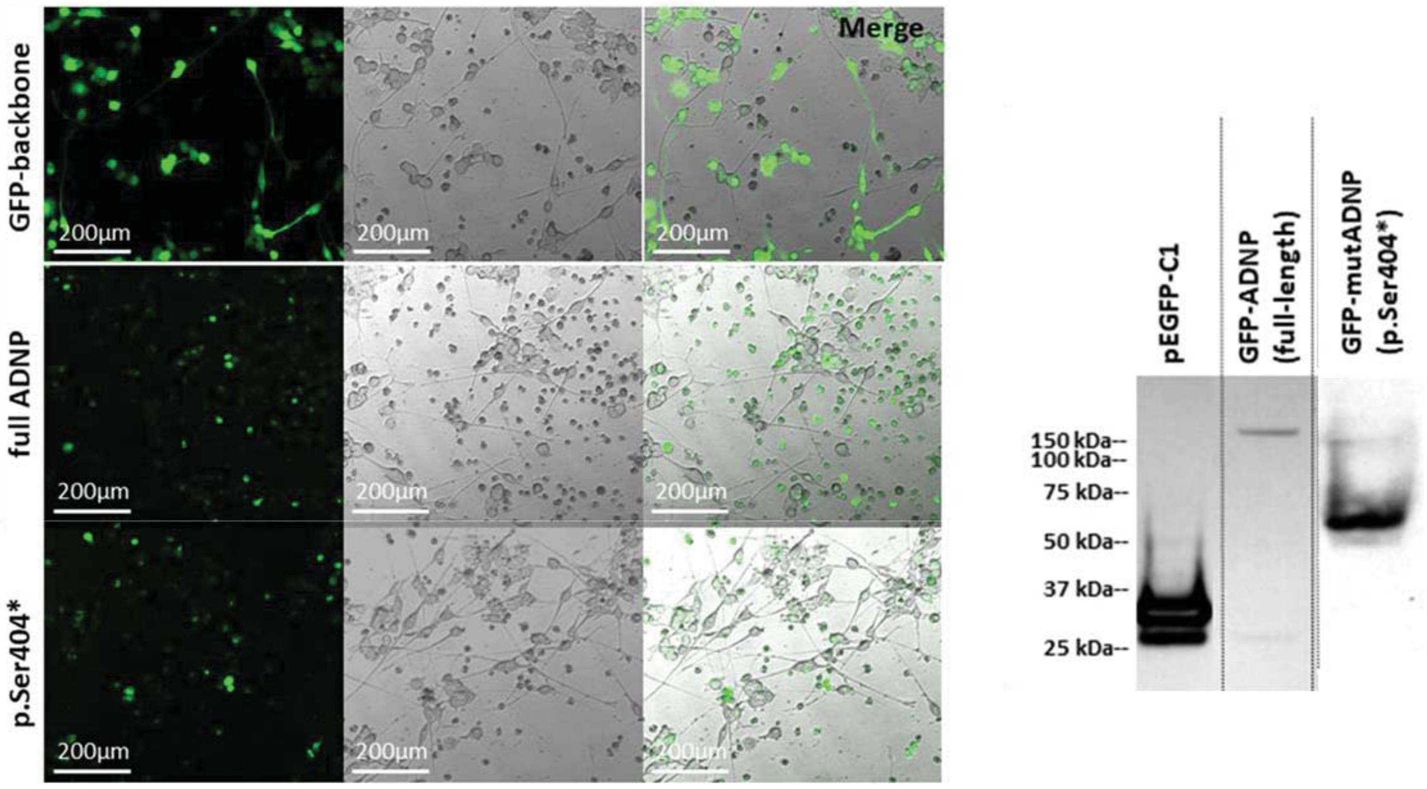

Figure S6A

Immunohistochemistry results of PSD95 and NMDAR1 in the hippocampal area

Immunohistochemical results for the case study hippocampal hillus and dentate gyrus.

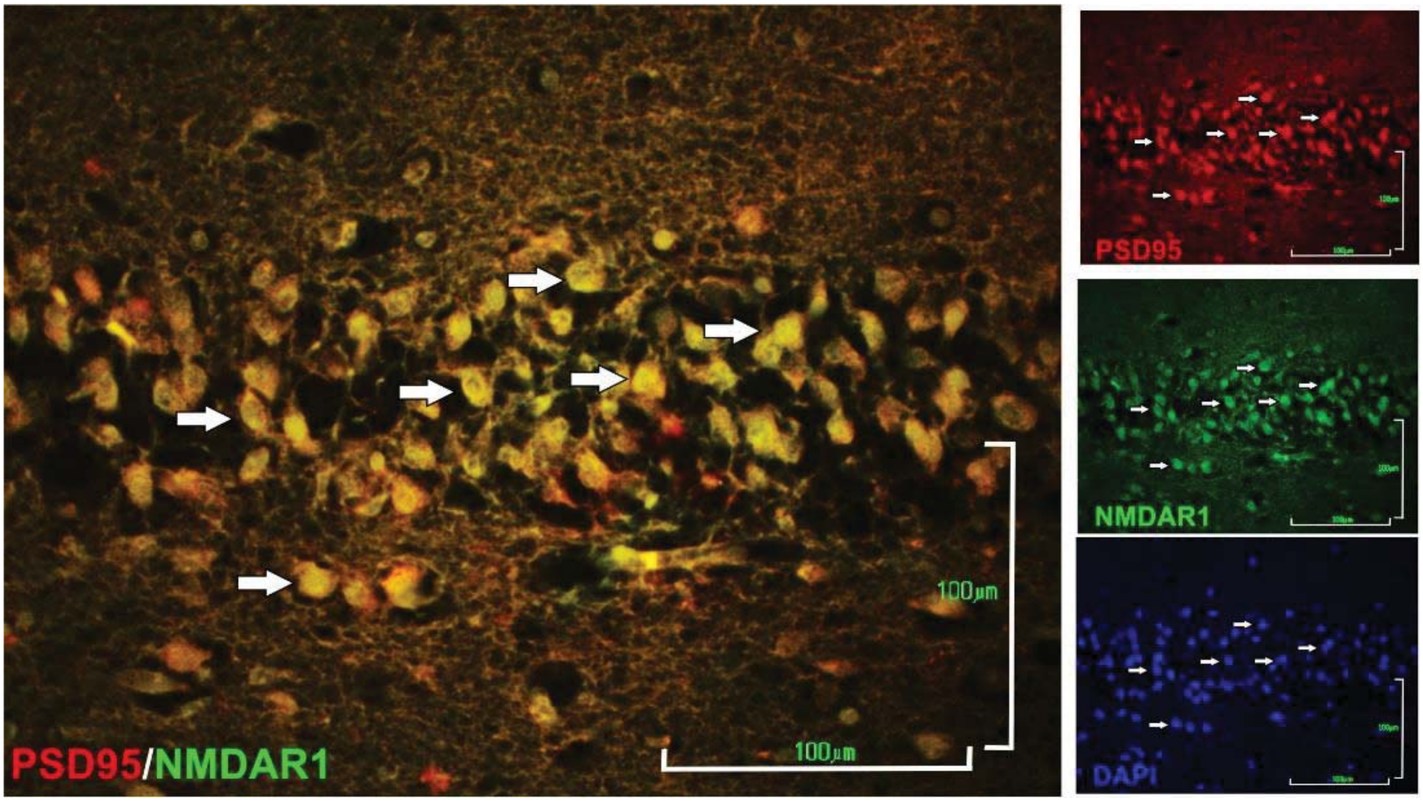

Figure S6B

Immunohistochemistry results of PSD95 and NMDAR1 staining in the hippocampal area of a control subject

The hippocampal block did not contain the granule cell layer of the CA1-4 or the dentate gyrus. Hippocampal hillus a control subject is shown.

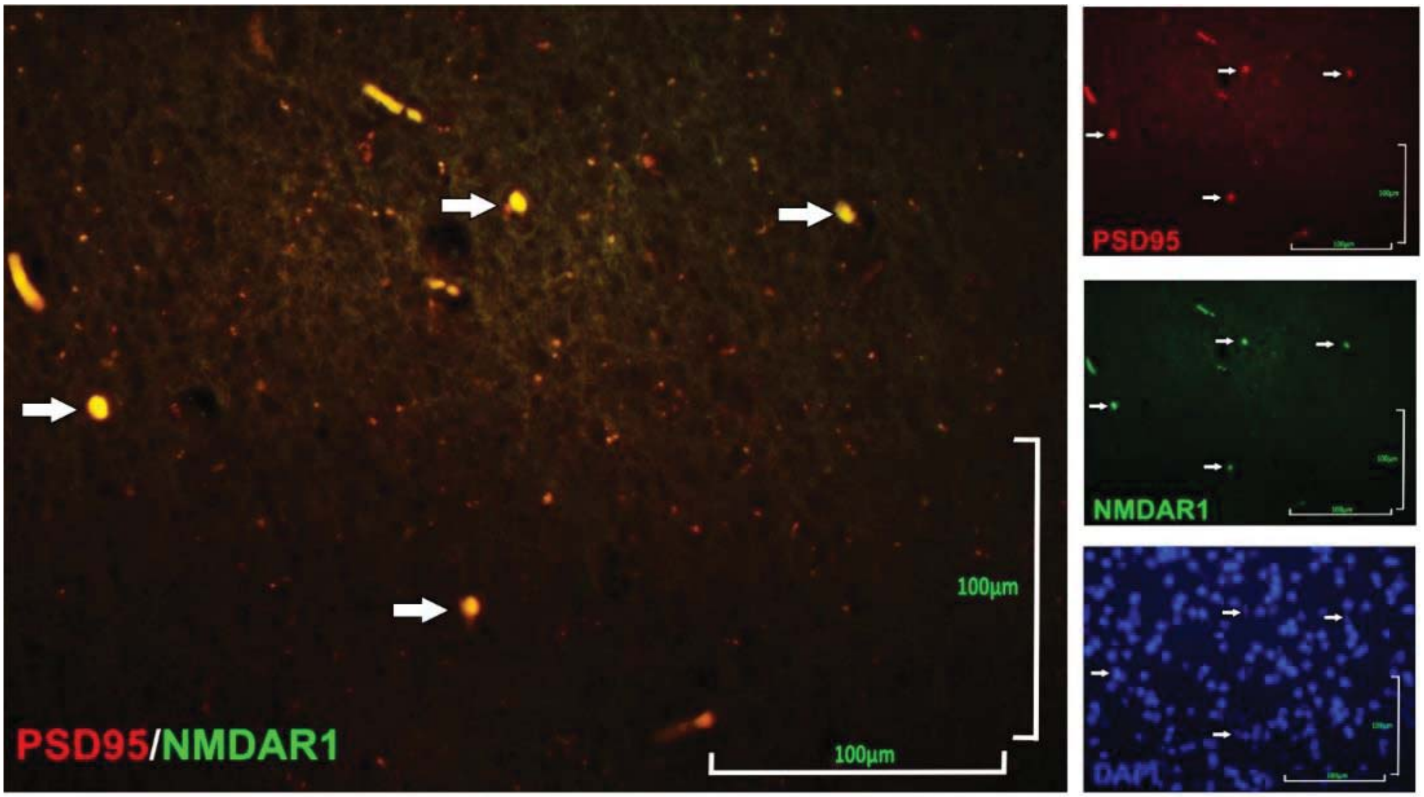

Figure S7

Spearman correlation test between datasets

For each gene, pairwise Spearman correlation coefficients (left) and p-Values (right) were computed between each pairs of datasets (ADNP syndrome, HPA, and GTEx) indicated at the bottom of the heatmaps. The vectors' sizes are the number of tissues having expression in both datasets. Correlation coefficients and p-Values are indicated within the cells.

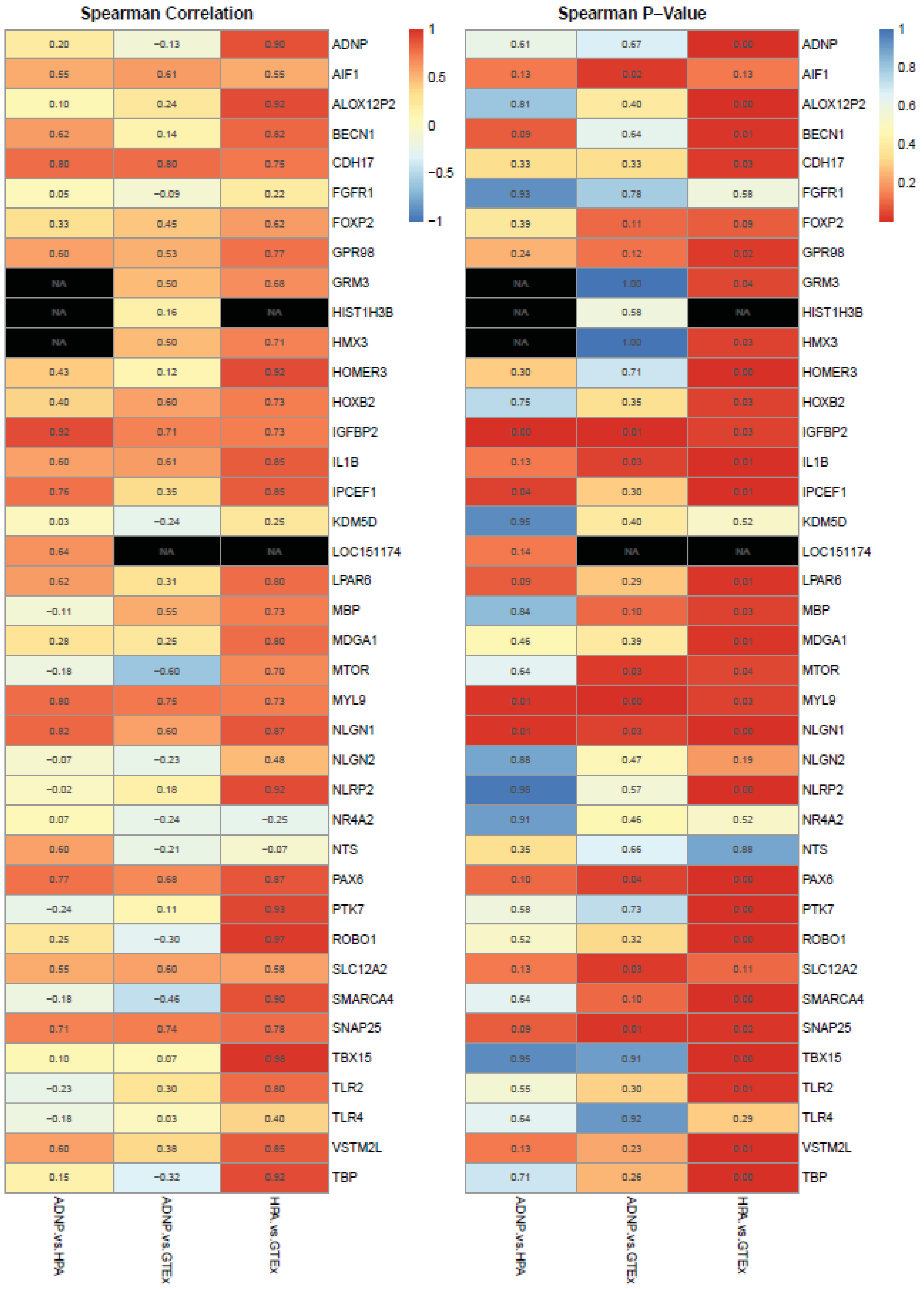
Spearman Correlation
Spearman P−Value
ADNP
AIF1
ALOX12P2
BECN1
CDH17
FGFR1
FOXP2
GPR98
GRM3
HIST1H3B
HMX3
HOMER3
HOXB2
IGFBP2
IL1B
IPCEF1
KDM5D
LOC151174
LPAR6
MBP
MDGA1
MTOR
MYL9
NLGN1
NLGN2
NLRP2
NR4A2
NTS
PAX6
PTK7
ROBO1
SLC12A2
SMARCA4
SNAP25
TBX15
TLR2
TLR4
VSTM2L
TBP
ADNP.vs.HPA
ADNP.vs.GTEx
HPA.vs.GTEx
1
0.5
0
−0.5
−1
0.8
0.6
0.4
0.2

Legends to the External Tables:

Table S1

GSE8126 dataset and log-fold change (LFC) values

We computed the LFCs between the three mutated ADNP samples against the control sample (Methods). We used the computed LFCs as the gene ranking metric for performing enrichment analysis using GSEA (Methods).

Table S2

GSEA enrichments results

We applied the GSEA on the LFCs from Table S1 to find enrichments in the following gene-sets: KEGG and Reactome pathways, GO biological process (BP), GO molecular function (MF), and GO cellular component (CC). Please see 'Methods' section for further details.

Table S3

Gene expression values of ADNP syndrome, HPA, and GTEx datasets

We collected two control datasets: HPA and GTEx, focusing on the expression values of 38 selected genes (Table 1 & 2), and the housekeeping gene, TBP (Methods).

Table S4

List of genes and primers selected for qPCR analysis

References

1. Amram N, Hacohen-Kleiman G, Sragovich S, et al. Sexual divergence in microtubule function: the novel intranasal microtubule targeting SKIP normalizes axonal transport and enhances memory. *Molecular psychiatry.* Jan 19 2016.
2. Ivashko-Pachima Y, Hadar A, Grigg I, et al. Discovery of autism/intellectual disability somatic mutations in Alzheimer's brains: mutated ADNP cytoskeletal impairments and repair as a case study. *Molecular psychiatry.* Oct 30 2019.
3. Ivashko-Pachima Y, Sayas CL, Malishkevich A, Gozes I. ADNP/NAP dramatically increase microtubule end-binding protein-Tau interaction: a novel avenue for protection against tauopathy. *Molecular psychiatry.* Sep 2017;22(9):1335-1344.
4. Gozes I, Van Dijck A, Hacohen-Kleiman G, et al. Premature primary tooth eruption in cognitive/motor-delayed ADNP-mutated children. *Translational psychiatry.* Jul 4 2017;7(7):e1166.
5. Schindelin J, Arganda-Carreras I, Frise E, et al. Fiji: an open-source platform for biological-image analysis. *Nature methods.* Jun 28 2012;9(7):676-682.
6. Rapsomaniki MA, Kotsantis P, Symeonidou IE, Giakoumakis NN, Taraviras S, Lygerou Z. easyFRAP: an interactive, easy-to-use tool for qualitative and quantitative analysis of FRAP data. *Bioinformatics.* Jul 1 2012;28(13):1800-1801.
7. Koulouras G, Panagopoulos A, Rapsomaniki MA, Giakoumakis NN, Taraviras S, Lygerou Z. EasyFRAP-web: a web-based tool for the analysis of fluorescence recovery after photobleaching data. *Nucleic acids research.* Jul 2 2018;46(W1):W467-W472.
8. Gozes I, Van Dijck A, Hacohen-Kleiman G, et al. Premature primary tooth eruption in cognitive/motor-delayed ADNP-mutated children. *Translational psychiatry.* Feb 21 2017;7(2):e1043.
9. Hacohen-Kleiman G, Sragovich S, Karmon G, et al. Activity-dependent neuroprotective protein deficiency models synaptic and developmental phenotypes of autism-like syndrome. *The Journal of clinical investigation.* Nov 1 2018;128(11):4956-4969.
10. Livak KJ, Schmittgen TD. Analysis of relative gene expression data using real-time quantitative PCR and the 2(-Delta Delta C(T)) Method. *Methods.* Dec 2001;25(4):402-408.
